# Confronting the Challenges in Lithium Anodes for Lithium Metal Batteries

**DOI:** 10.1002/advs.202101111

**Published:** 2021-07-01

**Authors:** Qingyu Wang, Bin Liu, Yuanhao Shen, Jingkun Wu, Zequan Zhao, Cheng Zhong, Wenbin Hu

**Affiliations:** ^1^ Key Laboratory of Advanced Ceramics and Machining Technology (Ministry of Education) Tianjin Key Laboratory of Composite and Functional Materials School of Materials Science and Engineering Tianjin University Tianjin 300072 China; ^2^ Joint School of National University of Singapore and Tianjin University International Campus of Tianjin University Binhai New City Fuzhou 119077 China

**Keywords:** coulombic efficiency, cyclic performance, high energy density, lithium anodes, lithium metal batteries, practical application

## Abstract

With the low redox potential of −3.04 V (vs SHE) and ultrahigh theoretical capacity of 3862 mAh g^−1^, lithium metal has been considered as promising anode material. However, lithium metal battery has ever suffered a trough in the past few decades due to its safety issues. Over the years, the limited energy density of the lithium‐ion battery cannot meet the growing demands of the advanced energy storage devices. Therefore, lithium metal anodes receive renewed attention, which have the potential to achieve high‐energy batteries. In this review, the history of the lithium anode is reviewed first. Then the failure mechanism of the lithium anode is analyzed, including dendrite, dead lithium, corrosion, and volume expansion of the lithium anode. Further, the strategies to alleviate the lithium anode issues in recent years are discussed emphatically. Eventually, remaining challenges of these strategies and possible research directions of lithium‐anode modification are presented to inspire innovation of lithium anode.

## Graphical abstract

The improvement of lithium anodes plays a great role in developing lithium metal batteries with high energy density. With the aim of enlighting the future directions of the researches on lithium anodes, the challenges and progress in the field of lithium anodes in recent years are presented.

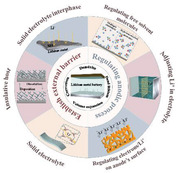

## 1 Introduction

**Scheme 1:**
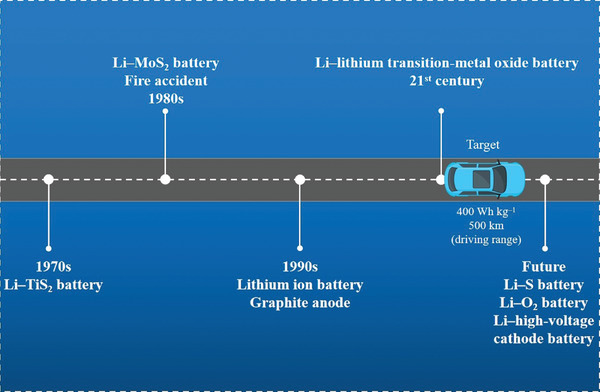
Schematic diagram of the development history of lithium metal batteries.

The demand for electric vehicles with long driving range has put forward higher requirements for the energy density of batteries.^[^ ^1^ ^]^ Lithium has long received much attention as a promising anode material. The interest in this alkali metal has arisen from its lowest redox potential of −3.04 V (vs SHE) and ultrahigh theoretical capacity of 3862 mAh g^−1^ of lithium anode; thus lithium metal batteries (at least 440 Wh kg^−1^)^[^ ^2^, ^3^, ^4^ ^]^ are considered as one of the most hopeful high energy density batteries. Particularly, lithium metal batteries based on high‐voltage cathodes, which are NCA (LiNi_0.8_Co_0.15_Al_0.05_O_2_), NCM811 (LiNi_0.8_Co_0.1_Mn_0.1_O_2_), and LNP (LiNiPO_4_), offer rather high energy densities (>550 Wh kg^−1^ and >1400 Wh L^−1^), with Li//LNP battery as the champion (618 Wh kg^−1^ and 1541 Wh L^−1^).^[^ ^5^ ^]^ During charging and discharging, lithium metal plating/stripping occurs on the anode, respectively. However, because of the intrinsic properties of lithium metal, lithium anode has many notorious problems in the process of charging/discharging: The formation of dendrites, the corrosion of lithium, dead lithium, and volume expansion. These problems will lead to severe capacity loss and even explosion of lithium metal batteries after long operation.^[^ ^6^, ^7^ ^]^ The early exploration of lithium metal batteries concentrated on the reversible lithium anodes, but the above issues have been hindering the practical application of lithium batteries.^[^ ^8^, ^9^ ^]^ Especially when the first commercialized lithium‐ion battery appeared in the 1990s,^[^ ^10^ ^]^ lithium metal anode was replaced by graphite, and lithium metal batteries seemed to fell into a trough. Nevertheless, as the energy density limitation (299 Wh kg^−1^) of traditional lithium‐ion batteries based on graphite anode cannot meet the demand for driving range of electric vehicles (500 km), researchers refocus on lithium anodes.^[^ ^11^ ^]^ Advanced in situ or ex situ characterization are used to reveal the failure mechanism of lithium metal anode and a variety of strategies have been proposed to alleviate issues of lithium metal. There is a gradual revival of lithium metal batteries.^[^ ^4^ ^]^ And to pursue higher energy density, future lithium metal batteries will be developed in the direction of high‐voltage cathodes, lithium sulfur batteries, and lithium oxygen batteries.^[^ ^11^, ^12^, ^13^, ^14^ ^]^ The research history of lithium metal batteries is shown in **Scheme** **1**.

After putting so much effort, the research on lithium metal anode has made great progress. It is generally believed that uneven deposition/dissolution,^[^ ^15^ ^]^ high reactivity of lithium, and uncontrollable volume expansion of lithium lead to a series of problems. Factors such as small battery pressure and the accumulation of LiH have also been demonstrated as the cause of lithium anode's failure under practical conditions (low N/P ratio, lean electrolyte and pouch battery system).^[^ ^16^, ^17^ ^]^ Additives, artificial solid electrolyte interphase (SEI), solid electrolyte, and 3D anode hosts, etc., are proposed to solve the lithium anode problems. Among them, the dual‐salt and tri‐salt electrolyte systems play synergistic effect in building stable SEI.^[^ ^18^ ^]^ The design of the organic/inorganic hybrid solid electrolyte and the electrolyte/electrode interface layer improve the contact between the solid electrolyte and the lithium anode.^[^ ^19^, ^20^ ^]^ A variety of methods guiding Li^+^ (such as, lithiophilic sites, gradient electrical conductivity, reconstructed lattice plane) contribute to the uniform and compact deposition of lithium in the 3D host.^[^ ^21^, ^22^, ^23^ ^]^ Compared with the concentrated mono‐salt, the concentrated dual‐salt effectively improves the ion conductivity of the high‐concentration electrolyte (HCE) system.^[^ ^24^ ^]^ Although the problem of lithium anode has been alleviated to some extent, to our knowledge, there is still a gap between the performances of present lithium metal batteries and practical application. Calculation and simulation, such as, molecular dynamic simulation and phase field model, are used to propose new directions for the improvement of host materials, electrolyte formulations, etc.^[^ ^25^, ^26^ ^]^

In this review, advanced studies on lithium anode in lithium metal batteries are discussed. Strategies in this paper are mainly divided into two categories: a) Establish an external barrier. Robust SEI, rigid solid electrolyte, and insulative host material are discussed in this section. b) Regulate the anode process. Regulating electrons/Li^+^ on the anode surface, adjusting Li^+^ in the electrolyte, reducing the free solvation in the electrolyte are contained in this part. Besides, some novel methods, such as the sandwich separator, are also summarized in this paper. Advanced simulation and calculation are also reviewed in this review to guide further experiments. Eventually, remaining challenges of these strategies and further developments of lithium‐anode modification are presented for designing stable anode in lithium metal battery. This work aims at illustrating the development in this field and thus inspiring innovation of lithium anode.

## 2 Mechanism and Challenges of Lithium Metal Batteries

### 2.1 Working Mechanism of Lithium Metal Batteries

This review is limited in the range of rechargeable lithium metal battery, and for brief‐expression, the following “lithium metal battery” refers to “rechargeable lithium metal battery.” Using the lithium metal as anodes, various lithium metal batteries have the same anodic reaction: Simple electrochemical dissolution/deposition processes of the lithium metal. Simultaneously, Li^+^ is consumed by the cathodic reaction. According to the types of cathode materials, lithium metal batteries can be divided into three primary categories: Lithium/lithium intercalation compound batteries, lithium/O_2_ batteries, and lithium/sulfur batteries. The reaction principle of lithium metal battery in the charge and discharge process is described as follows:

Lithium metal battery with intercalation cathode: (take the example of metal oxides Li_1‐_ *_x_*MO_2_)_:_^[^ ^27^, ^28^ ^]^

(1) $$\mathrm{Anode}\;\mathrm{reaction}:\;x\mathrm{Li}=xLi^{+}+xe^{-}$$

(2) $$\mathrm{Cathode}\;\mathrm{reaction}:\;Li_{1-x}MO_{2}+xLi^{+}+xe^{-}=\mathrm{LiM}O_{2}$$

Li‐S battery:^[^ ^27^, ^28^ ^]^

(3) $$\mathrm{Anode}\;\mathrm{reaction}:\;16\mathrm{Li}=16Li^{+}+16e^{-}$$

(4) $$\mathrm{Cathode}\;\mathrm{reaction}:\;S_{8}+16Li^{+}+16e^{-}=8Li_{2}S$$

Li‐O_2_ battery_:_^[^ ^27^, ^28^, ^29^ ^]^

(5) $$\mathrm{Anode}\;\mathrm{reaction}:\;2\mathrm{Li}=2Li^{+}+2e^{-}$$

(6) $$\mathrm{Cathode}\;\mathrm{reaction}:\;1/2O_{2}+H_{2}O+2e^{-}=2OH^{-}$$

(7) $$\mathrm{or}\;2Li^{+}+2e^{-}+O_{2}=Li_{2}O_{2}$$

The formation of the SEI is one of the most significant procedures in anode process and needs to be noted. Due to the very negative redox potential of lithium, the components in the electrolyte are easily reduced by lithium metal,^[^ ^30^ ^]^ and the reduction products cover on the anode surface and become the composition of SEI. In general, the passivation SEI can prevent further corrosion of the electrode material. However, due to the uneven deposition/dissolution of lithium metal, the SEI layer on lithium surface is much less stable than that in lithium‐ion batteries.^[^ ^8^, ^27^ ^]^ During the operation of the lithium metal battery, the inhomogeneity of anode accumulates and causes large stress under the SEI. And the SEI will be broken due to the release of stress.^[^ ^15^ ^]^ Lithium newly exposed in electrolyte reacts with the electrolyte to form a new, uneven SEI, and the process mentioned above repeats on the new SEI, which induces the incessant consumption of electrolytes and gradual increment of the battery internal resistance, and then conduces to low coulombic efficiency (CE) and rapid degradation of the capacity. Moreover, dendrites will also grow from the cracks on the SEI surface.^[^ ^15^ ^]^ Therefore, the stability of SEI is closely related to the stability of lithium anode.^[^ ^27^ ^]^

### 2.2 The Failure of the Lithium Anode

Before the lithium metal battery can develop into a feasible technology, tough challenges must be confronted, the greatest of which are batteries’ stability and safety.^[^ ^4^ ^]^ Both of these problems are closely related to lithium anode problems: Dendrite, dead lithium, corrosion, and volume expansion of lithium.

#### 2.2.1 Lithium Dendrite

**Figure 1:**
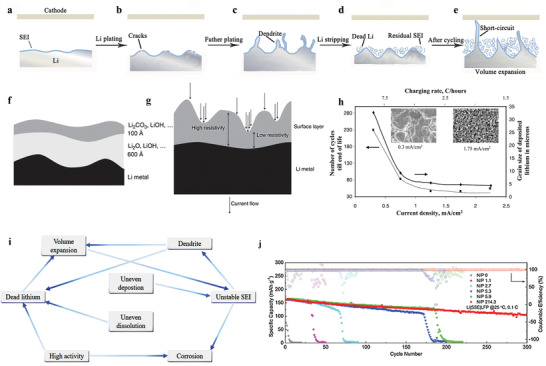
a–e) Schematic illustration of lithium anode's failure process. f) Schematic illustration of native surface layer on lithium and g) schematic illustration of uneven current distribution on the surface of lithium anode. f,g) Reproduced with permission.^[^ ^27^ ^]^ Copyright 2009, Elsevier. h) Typical behavior of lithium anode in Li‐Li*_x_*MnO_2_ AA batteries at different charging rates. The average size of lithium is associated with the charging rates. Corresponding SEM micrographs of lithium grains at different charging rates are also shown. Reproduced with permission.^[^ ^55^ ^]^ Copyright 2002, Elsevier. i) The correlations among anode issues. j) Cyclic performance of lithium metal batteries at various N/P ratios. Reproduced with permission.^[^ ^56^ ^]^ Copyright 2021, Wiley‐VCH.

Lithium tends to deposit in the form of dendrites on the anode current collector. When the dendrites grow big enough to pierce the separator and cause short circuit, large current passes through thin dendritic connection, which rapidly generates heat and results in the safety risks of fire and explosion.^[^ ^31^ ^]^ This is a severe problem to restrict the commercialization of lithium metal battery because the safety of the battery on the user's side must be ensured. Some studies have revealed the growth process of lithium dendrites (**Figure** **1**a–c): Lithium deposits unevenly and the inhomogeneity increases with cycles, creating large stresses under the SEI. Consequently, the SEI is broken due to the release of stress. Lithium grows from the cracks and forms the initial dendrite, which will gradually grow until the short circuit occurs in the battery.^[^ ^15^ ^]^ Dendrite formation is the result of the uneven deposition of lithium, which can be attributed to both thermodynamic and kinetic factors.

Among metallic anodes, the surface energy density of the lithium metal is lowest.^[^ ^32^ ^]^ This property favors the growth of dendrites.^[^ ^14^, ^33^ ^]^ Because of this thermodynamic tendency, dendrites cannot be thoroughly eliminated. From the kinetic point, many factors can lead to lithium dendrites: The surface states of lithium, anodic electrical field, etc. On the surface of pristine lithium, there is an uneven native layer, consisting of Li_2_O, LiOH, Li_2_CO_3_ (Figure 1f), and surface defects, which forms during the production process of lithium foil.^[^ ^34^, ^35^ ^]^ When the lithium is introduced into battery and immersed in electrolyte, generally, the formed SEI on the surface of the lithium metal is not so homogeneous.^[^ ^27^ ^]^ These uneven surface states of lithium will lead to uneven conductivity and current density on lithium surface (Figure 1g) and, eventually, result in the uneven plating of lithium. The uneven electrical field caused by the concentration gradient of ions and the unsteady state of electrolyte concentration is also an explanation of dendrite growth. Chazalviel^[^ ^36^ ^]^ found that the transport of Li^+^ is more difficult than that of anions because of the abundant solvent molecules in the solvent sheath of Li^+^. Consequently, the concentration of the anions adjacent to cathode rapidly comes down to zero, provoking a positive space charge area. The space charge domain generates the local electric field, instigating the growth of the dendrites. Electrolytes with better ionic conductivity and decreased anion mobility suppress the nucleation of the dendrites via alleviating anion depletion nearby the electrode/electrolyte interface.^[^ ^37^ ^]^ The charge/discharge protocol of batteries also affects the formation of lithium dendrites.^[^ ^15^, ^38^ ^]^ In live scanning electron microscope observations, Dollé et al.^[^ ^15^ ^]^ noticed that lithium evolved from mossy to needle like as the current density increases. Besides, according to the calculation based on the mechano‐electrochemical phase field model, within a certain range, the lithium deposited on the anode was smoother when the battery subjected to higher pressure. It revealed a reason that the cyclic performance of the practical pouch batteries were worse than that of the coin batteries: The pressure undertaken by lithium anode of the pouch battery was lower than that of coin battery. However, too high pressure possibly caused the dendrites to break from the root, resulting in the loss of capacity. Therefore, appropriate pressure was conducive to obtaining lithium metal batteries with prominent performance.^[^ ^16^ ^]^

#### 2.2.2 Dead Lithium

Lithium losing contact with the electrode cannot participate in the electrode reaction anymore, is so‐called dead lithium. Dead lithium means the irreversible capacity loss of the batteries.^[^ ^39^ ^]^ It is reported that the CE of lithium deposition/dissolution in non‐aqueous electrolytes is merely <99.2% owing to uncontrolled dendrites and dead lithium.^[^ ^40^ ^]^ It is necessary to understand the formation process of dead lithium. As Figure 1d shown, dead lithium originates from the broken dendrite, and the break often occurs at thin necks of dendrites, which results from that thin necks of dendrites with larger curvature tend to accumulate larger electron densities, and subsequently exhibit faster lithium electro‐dissolution rates.^[^ ^32^, ^41^ ^]^ After the break of the dendrite, the freshly exposed lithium surface is corroded quickly by electrolyte and SEI with poor electronic conductivity forms around broken lithium. The broken lithium losses electrical connection with the anode and could not participate in the subsequent reaction. Therefore, it is easy to form dead lithium in the case of a large number of dendrites with slim structures. To a certain extent, the factors affecting the formation of dendrites will also affect the formation of dead lithium, such as, charge/discharge protocol,^[^ ^42^, ^43^ ^]^ stress,^[^ ^44^ ^]^ etc. Another situation is that for a porous current collector with a larger pore size, due to uneven current distribution, the lithium metal located in the center of the pore easily loses connection with the porous framework, forming “dead lithium.” Therefore, it is significant to control the pore size of the porous current collector.^[^ ^45^ ^]^

#### 2.2.3 Corrosion of Lithium Anode

Because of highly negative redox potential, lithium spontaneously reacts with electrolytes and form the SEI.^[^ ^46^ ^]^ Due to the repeating break of unstable SEI during cycling, the fresh lithium is exposed to the electrolyte and react with the electrolyte over and over again. The continuous depletion of electrolyte and the electrochemical corrosion of lithium metal will induce low CE and capacity decay.^[^ ^47^, ^48^ ^]^ The electronic structure, viscosity of electrolyte, and stability of SEI are three factors that can affect the reaction between lithium and electrolyte: The electrochemical reduction potential of the material connects closely to its lowest unoccupied molecular orbital (LUMO) energy. Generally, a low LUMO energy of the material indicates a high reduction potential.^[^ ^48^ ^]^ Thus an electrolyte with a higher LUMO is less likely to react with lithium. For example, ethers can realize better cyclic performance in the lithium metal battery than easter because of its higher LUMO.^[^ ^49^ ^]^ The viscosity of electrolyte also affects the corrosion rate of lithium. According to previous reports, in contrast to propylene carbonate and ethylene carbonate, low‐viscosity dimethoxyethane and tetrahydrofuran are less stable against lithium.^[^ ^27^ ^]^ Robust SEI can prevent exposure of fresh lithium, which is a direct factor to influence the corrosion of lithium. SEI is derived from the reduction of electrolyte on the surface of lithium metal, and its composition and properties are closely related to that of electrolyte. For example, in ordinary ethylene carbonate electrolyte, an unstable SEI is formed on the surface of the lithium metal battery.^[^ ^50^ ^]^ With the fluorine‐substituted cyclic carbonates electrolyte, the surface of lithium anode produces a LiF‐rich and chemically stable SEI.^[^ ^51^ ^]^ As a result, lithium corrosion can be alleviated by adjusting the electrolyte formula and improving the stability of SEI.

#### 2.2.4 The Expansion of the Anodic Volume

The loose structure of dendrites will cause the expansion of anode volume (Figure 1e). In the process of lithium's plating/stripping, the formation rate of the SEI cannot match the expansion of lithium anode. Ultimately, the SEI fractures, leading to the growth of dendrites.^[^ ^52^ ^]^ A vicious cycle between dendrite and volume expansion forms, which is a grand challenge. Moreover, this volume expansion induces the disruption of battery interfaces and leads to the decay of the battery's performance. For example, in solid state lithium metal batteries, the separation between the solid electrolyte and electrode interface will lead to the continuous increase of cell resistances, and cause the failure of battery finally. Besides, the stacked dead lithium also contributed to the increase in anode thickness (Figure 1e). After dozens of cycles, the thickness of the lithium anode can increase from tens to hundreds of micrometers.^[^ ^53^ ^]^ The stacked dead lithium hinders the transport of lithium ions and, hence, leads to the voltage losses.^[^ ^54^ ^]^ In practical lithium metal batteries, the insulting and pulverized LiH has been proved to be one of the reasons for anodic expansion and the failure of lithium metal battery, which results from the side reaction between lithium and the H_2_ in the battery. Xu et al.^[^ ^17^ ^]^ studied practical battery with an ultra‐thin lithium anode and a high‐load cathode (LiCoO_2_). It was found that in anodic surface, the proportion of LiH in the anodic byproducts increases dramatically (from 0.74% to 16.55%) after 20 cycles. And the amount of LiH on the surface of anode was negatively correlated with the lifespan of the batteries with different electrolyte systems.

It is important to note that some researchers think that the corrosion of the lithium, the consumption of electrolyte and the porous anode with high resistance are the main reasons for the failure of lithium metal battery rather than dendrite. Aurbach et al.^[^ ^55^ ^]^ proposed that the lithium metal tended to deposit into small grains upon fast charging (Figure 1h). Because of the increased specific surface area of lithium metal, the corrosion of lithium aggravated, contributing to the failure of the battery. Lu et al.^[^ ^50^ ^]^ also found no evidence of separator pierced by dendrites in the failure battery, even at a high current density (1.5 mA cm^−2^). And instead, the expansive areas of lithium/electrolyte interface at high current density prompted the formation of more SEI components, inhibiting the contacts among the lithium grains and causing a porous structure. The increasing resistance of this porous interphase was considered the true reason for the eventual failure of the lithium metal battery. Therefore, further research needs to be carried out to investigate the true failure causes of lithium metal batteries.

### 2.3 The Correlation among Anode Issues

The issues of lithium anode described above are not independent with each other. And the correlations among these issues are illustrated in Figure 1i. The uneven deposition of lithium metal leads to the formation of the stress field under the SEI, and the release of stress causes the crack of SEI. Lithium metal grows from the crack, forming whiskers (dendrites). The unstable SEI and high activity of lithium lead to the severe corrosion of lithium, generating the loss of lithium. Dendrites break because of the uneven dissolution of lithium, and the high activity of lithium insulates them from the matrix, resulting in the formation of dead lithium. After continuous cycles, due to the stack of loose dendrites and dead lithium, the anode gradually expands.^[^ ^4^ ^]^ In turn, the volume fluctuation of the anode destroys the SEI and promotes the formation of dendrites. The uneven deposition is the origin of so many issues. The unstable SEI is the direct result of uneven deposition. Therefore, the strategies of promoting uniform lithium deposition and improving the stability of SEI can solve these issues relatively comprehensively.

### 2.4 The Gap between Coin and Practical Lithium Metal Battery

In view of the loss of lithium during cycling, the N/P (negative/positive electrode capacity) ratio and the amount of electrolyte are considered by the researchers. According to the report of Chen et al.,^[^ ^56^ ^]^ the cyclic performance of lithium metal batteries was significantly improved with increased N/P ratio (Figure 1j). Excess electrolyte will also prolong batteries’ lifespan by reducing the resistance of ionic transmission. However, considering the cost and energy density of the battery, the amount of electrolyte in a practical battery is an order of magnitude smaller than that in the coin battery tested in the lab.^[^ ^57^ ^]^ According to Liu's report,^[^ ^58^ ^]^ the high‐energy battery of 300 Wh kg^−1^ requires a thin lithium anode of 50 µm. And the battery of 350 Wh kg^−1^ needs the lean electrolyte amount of about 3 g (Ah)^−1^, while the 75 µL electrolyte commonly used in CR2032 coin battery is equal to an electrolyte/capacity ratio of about 70 g (Ah)^−1^. The value is far beyond the practical standard of 3 g (Ah)^−1^. Albertus et al.^[^ ^59^ ^]^ also proposed that the thickness of the lithium sheet should be controlled (<30 µm) to help identify the soft short phenomenon in symmetrical lithium batteries. Moreover, compared with the coin battery, the practical pouch battery has a larger reaction area and the unevenness of the electrode's surface is more obvious. This results in faster failure of lithium anode. Under the same conditions of test, the lifespan of the pouch lithium metal battery is only 1/3–1/10 of that of the coin battery.^[^ ^60^ ^]^ As mentioned earlier, due to the lack of space constraint derived from the rigid steel shell, the pressure undertaken by lithium anode in pouch battery is less than that in coin battery, which induces larger ion‐transfer resistance.^[^ ^57^ ^]^ The absence of space confinement also leads to the proliferation of lithium dendrites.^[^ ^16^ ^]^ The gap between practical lithium metal batteries and laboratory‐grade batteries is obvious. To get closer to practical applications, some studies have begun to use low N/P ratios, lean electrolyte, and pouch battery system in the tests of lithium metal batteries, which will be reviewed in the following text.

## 3 Strategies to Solve Issues of the Lithium Anode

Most of the previous studies have demonstrated that the lithium anodes can be stabilized by establishing external barrier and regulating anode process, which prevents the issues such as dendrite, dead lithium, corrosion, and expansion suffered by Li.^[^ ^50^ ^]^ The external barrier can be established by forming a robust SEI on the surface of the anode, using rigid solid electrolyte, or confining lithium in the host. While regulating the anode process includes regulating electrons/Li^+^ on the anode surface, adjusting Li^+^ in the electrolyte, and altering the solvation sheath of Li^+^. The modification process can be achieved by altering components related to the stability of lithium anode, such as, the composition of the electrolyte, SEI, structure of anode, etc.

### 3.1 Establish an External Barrier

#### 3.1.1 Solid Electrolyte Interphase

A robust SEI not only effectively helps freshly deposited lithium avoid being exposed to electrolyte,^[^ ^61^ ^]^ but also inhibits dendrites grow.^[^ ^62^, ^63^ ^]^ Therefore, two schemes are used to obtain the ideal SEI: a) Using special electrolyte formulation to improve the physical and chemical properties of in situ SEI; b) preparing a desired ex situ SEI.

##### In Situ Solid Electrolyte Interphase

In situ SEI is naturally generated on the lithium surface during battery operation. In the formulation of electrolyte, additives, solvents, and salts all affect the properties of the in situ SEI. The SEI with favorable chemical composition could form by using proper electrolyte formulation. The conventional LiPF_6_/ethylene carbonate‐ethyl methyl carbonate (EC‐EMC) system has been demonstrated incompatible with lithium metal anode, and the lithium metal batteries fail because the porous phase with high resistance gradually form on the anode during the cycling.^[^ ^50^ ^]^ To address this issue, the fluorine‐substituted cyclic carbonates were developed to enhance the stability of lithium, which could increase the LiF content in SEI. LiF has high chemical stability against lithium, which benefits to protecting lithium from being corroded.^[^ ^51^ ^]^ According to the study of Su,^[^ ^64^ ^]^ compared with the EC‐based electrolyte, monofluoroethylene carbonate (FEC)‐based and trans‐difluoroethylene carbonate (DFEC)‐based electrolyte promoted compact SEI layer containing higher level of LiF forming on the anode, which was the result of these two fluorinated species’ decomposition. And the Li//NMC622 battery using DFEC‐based electrolyte delivered high CE of 99.95% (average 400–cycle CE) at a C/3 rate. In contrast, in the EC‐based electrolyte, the battery delivered a much lower CE of 98.35% (average 100–cycle CE) and failed after about 120 cycles.

**Figure 2:**
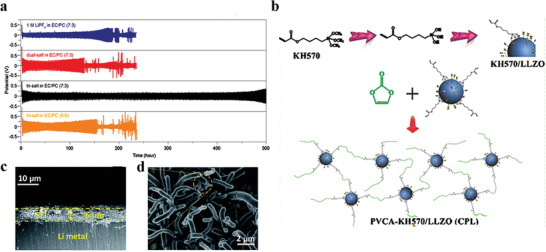
a) Cycling performance of Li||Li symmetric batteries with different electrolytes. The battery with tri‐salt electrolyte shows enhanced lifespan compared with the dual‐salt and mono‐salt cases. Reproduced with permission.^[^ ^68^ ^]^ Copyright 2019, American Chemical Society. b) Schematic illustration of the flexible polymer and inorganic superionic conductor network. Reproduced with permission.^[^ ^88^ ^]^ Copyright 2018, Wiley‐VCH. c,d) The SEM images of ex situ SEI prepared by immersing lithium in Mn(NO_3_)_2_‐containing carbonate electrolyte. Reproduced with permission.^[^ ^90^ ^]^ Copyright 2018, The Royal Society of Chemistry.

Since the surface chemistry of the lithium anode is strongly impacted by the reduction of the solute anions, lithium salts in non‐aqueous electrolytes play a key role in the CEs of lithium anodes.^[^ ^65^ ^]^ Because of the balanced properties, such as, less toxic, good ion conductivity, electrochemically stable, etc., LiPF_6_ is the most commonly used solute to date.^[^ ^66^, ^67^ ^]^ Nevertheless, LiPF_6_/EC‐based electrolyte has been demonstrated to induce unstable SEI.^[^ ^64^, ^68^ ^]^ Therefore, various salts are probed to obtain the ideal SEI. Recently, dual‐salt or tri‐salt systems, such as, lithium difluorophosphate‐lithium bis(oxalato)borate (LiDFP‐LiBOB),^[^ ^18^ ^]^ lithium bis(trifluoromethanesulfonyl)imide‐lithium bis(oxalato)borate (LiTFSI‐LiBOB),^[^ ^69^ ^]^ lithium bis(trifluoromethanesulfonyl)imide‐lithium bis(oxalato)borate‐lithium hexafluorophosphate (LiTFSI‐LiBOB‐LiPF_6_),^[^ ^68^ ^]^ become a thriving trend and the synergistic effect among different salts has been verified beneficial to form SEI with favorable components and proper proportion of these components. In a dual salt (LiDFP‐LiBOB) system, the Li//LiFePO_4_ battery operated stably for 300 cycles at the current density of 2 mA cm^−2^. The capacity retention remained at 95.4% and average CE was 99.8%. While in the ordinary electrolyte (LiPF_6_‐EC/DEC) system, the capacity decline occurred after only 70 cycles. This was attributed to the synergistic effect of the two salts: The decomposition of LiDFP generates LiF and P‐O species on the surface of the anode, improving the rigidity and ion conductivity of the SEI, respectively. Li_2_BO*_x_*, the decomposition products of LiBOB, contributed to suppressing the dissolution of the organic species in SEI and enhanced its flexibility.^[^ ^18^ ^]^ Jin et al.^[^ ^68^ ^]^ confirmed that lithium symmetric battery was more stable in a tri‐salt system (LiTFSI‐LiBOB‐LiPF_6_) than in LiPF_6_ or dual‐salt (LiTFSI‐LiBOB) systems (**Figure** **2**a), which was due to that tri‐salt system facilitated the formation of high‐quality SEI that contains sufficient both Li_2_CO_3_ and ROCO_2_Li. Highly stable Li_2_CO_3_ contributes to suppress the corrosion of lithium and ROCO_2_Li acts like the binder to maintain the integrity of SEI to achieve enhanced toughness. And strong LiF component was also detected through the whole formed SEI in tri‐salt system, which made the SEI heterogeneous and compact. In contrast, the SEI formed in the LiPF_6_ system contained little ROCO_2_Li, and the SEI formed in dual‐salt case only contained the LiF component close to the surface of SEI, but not deeply inside.

If the cost is considered, electrolyte additive is an economical strategy. Only a little additive significantly improves the performance of the anode. The low LUMO of the additives causes their preferential reduction before electrolyte on the surface of lithium, forming SEI with excellent properties.^[^ ^70^ ^]^ Common additives contain organic compounds (such as, vinylene carbonate,^[^ ^71^ ^]^ trimethylsilyl azide (TSA),^[^ ^63^ ^]^ 2‐(triphenylphosphoranylidene) succinic anhydride,^[^ ^72^ ^]^ tris(pentafluorophenyl)borane,^[^ ^70^ ^]^ fluoroethylene carbonate,^[^ ^73^ ^]^ thiourea,^[^ ^74^ ^]^ adiponitrile,^[^ ^75^ ^]^ and polydimethylsiloxane^[^ ^76^ ^]^), lithium salt (such as, LiPF_6,_^[^ ^77^ ^]^ LiPO_2_F_2_,^[^ ^78^ ^]^ LiF,^[^ ^51^ ^]^ and LiNO_3_^[^ ^79^, ^80^ ^]^), HF,^[^ ^51^ ^]^ and gas molecules (such as, SO_2_^[^ ^81^ ^]^). Among them, F‐rich, S‐rich, N‐rich chemicals have been extensively studied. Fluorinated species generate SEI with higher levels of dense LiF,^[^ ^68^ ^]^ physically suppressing dendrites. For instance, by adding diphenyl sulfone (DPS) and bis(4‐florophenyl) sulfone (BFS) in LiPF_6_/EC‐EMC‐DEC (diethyl carbonate) electrolyte, the specific capacity of Li//NMC battery didnot decay rapidly until 500 cycles at a high rate of 5C. According to the results of XPS, the amount of LiF in the SEI originated from these two additives was higher than that from the additive‐free electrolyte, which contributed to the excellent cyclic performance.^[^ ^82^ ^]^ However, the relatively poor ionic conductivity (10^−30^ S cm^−1^) of the LiF cannot satisfy the requirement for fast‐charging application.^[^ ^83^ ^]^ Therefore, the N‐rich or S‐rich chemicals were developed to produce highly conductive nitrides (such as, Li_3_N) or sulfide (such as, lithium sulfonates) on the surface of SEI. It is reported that the Li_3_N is one of the most conductive components of lithium‐ion conductivity (6.6 × 10^−4^ S cm^−1^) among diverse SEI components and that of lithium sulfonates is up to 10^−7^ S cm^−1^,^[^ ^84^, ^85^ ^]^ which promote reduced resistance of the SEI and improved reversibility of the lithium plating/stripping process.^[^ ^86^ ^]^ Using trimethylsilyl azide (TSA)‐added electrolyte, Li//FCG73 (Li[Ni_0.73_Co_0.10_Mn_0.15_Al_0.02_]O_2_) battery exhibited outstanding cyclic performance (CE ≈ 99.75% after 300 cycles) even at the high current density of 2 mA cm^−2^, while the Li//FCG73 battery in the ordinary electrolyte (1 m LiPF_6_ dissolved in EMC/FEC) failed after about 270 cycles. It was mainly because formed SEI in the modified electrolyte contained conductive Li*_x_*N (possibly Li_3_N) compounds, which was in favor of the ions’ transport under the large current density.^[^ ^63^ ^]^ Additives adsorbed on the anodic surface can also directly induce the formation of special lithium morphology. Luo et al.^[^ ^87^ ^]^ used caffeic acid (CA) as the electrolyte additive, which was adsorbed on the surface of lithium and polymerized in situ to form CA‐Li film. The SEM morphology of deposited lithium was spherical after 100 cycles. The DFT calculation indicated that the lithium atom on the polymer chain was chemically trapped and intimately contacted with other lithium atoms on adjacent monomers. This confinement constrained lithium atoms together for nucleation/growth and forming nanosphere. The dendrite‐free deposition of lithium generated distinguished cyclic lifespan, more than twice that of batteries without CA. However, although various electrolyte additives have been investigated, many studies explain the enhanced electrochemical properties by merely studying the decomposition products of the electrolyte additives. The lack of insightful theoretical guidance about the detailed reaction process is an obstacle to developing new additives.

Recent progress of in situ SEI is summarized in **Table** **1**. On the whole, these studies give lithium metal battery outstanding rate and cycle performance. The CE of lithium metal batteries in suitable electrolytic liquid systems even reach 99.75% (after 300 cycles), which is a relatively prominent strategy among numerous strategies to stabilize lithium anode. This indicates that the in situ SEI can effectively protect lithium metal, and this strategy has a broad prospect.

**Table 1:** Summary of recent studies on in situ SEI of the lithium anode

Material	Type	Overpotential for charging/discharging	CE	Ref.
DPS	Additive	<400 mV (360 h, symmetric battery, 1 mA cm^−2^, 2 mAh cm^−2^)	99.5% (500 cycles, Li//NMC, battery, 1C, –)	[82]
Thiourea (THU)	Additive	≈20 mV (1000 cycles, symmetric battery, 5.0 mA cm^−2^, 1.0 mAh cm^−2^)	Average CE = 98.5% (350 cycles, Li||Cu battery, 0.5 mA cm^−2^, 1.0 mAh cm^−2^)	[74]
C_6_H_8_N_2_	Additive	A few hundred mV (900 h, 450 cycles, symmetric battery, 1.8 mA cm^−2^, 1.8 mAh cm^−2^)	>99.5% (> 830 cycles, Li//FCG73 battery, 1.8 mA cm^−2^, 1.8 mAh cm^−2^)	[75]
Polydimethylsiloxane (PDMS)	Additive	≈100 mV (>1800 h, symmetric battery, 0.5 mA cm^−2^, 1.5 mAh cm^−2^)	>90% (140 cycles, Li||Cu battery, 1 mA cm^−2^, 2 mAh cm^−2^)	[76]
LiPF_6_+ LiTFSI‐LiBOB	Additive + dual salt	Unspecified overpotential (500 cycles, Li//NMC battery, 1.75 mA cm^−2^, –)	>99% (800 cycles, Li//NMC battery, 0.58 mA cm^−2^ for charge and 1.75 mA cm^−2^ for discharge, –)	[77]
DFEC	Solvent	15 mV (>1000 h, symmetric battery, 2 mA cm^−2^, –)	Average CE = 97.1% (10 cycles, Li||Cu battery, 1 mA cm^−2^, –)	[64]
LiNO_3_	Additive	220 mV (120 cycles, Li//LFP battery, 0.5C, –)	Average CE > 99.5% (after 300 cycles, Li//NCA battery, 0.5C, –)	[79]
TSA	Additive	≈100 mV (>1200 h, symmetric battery, 2.0 mA cm^−2^, 2.0 mAh cm^−2^)	≈99.75% (300 cycles, Li//FCG73 battery, 2.0 mA cm^−2^, 4.0 mAh cm^−2^)	[63]
LiTFSI‐LiBOB‐LiPF_6_	Tri‐salt system	152 mV (400 h, symmetric battery, 1 mA cm^−2^, 1 mAh cm^−2^)	Average CE = 99.7% (400 cycles, Li//NMC battery, 0.83 mA cm^−2^, –)	[68]

##### Ex Situ Solid Electrolyte Interphase

The ex situ SEI is a protective layer artificially prepared on the lithium surface before the battery is assembled. The ex situ SEI fabricated by polymer coating,^[^ ^88^ ^]^ sputtering,^[^ ^89^ ^]^ immersing in solvent,^[^ ^90^, ^91^ ^]^ dip‐casting,^[^ ^92^ ^]^ is conducive to optimize the electrochemical properties of the lithium anode. Compared with the in situ SEI, ex situ SEI has great attraction because of its controllable composition, thickness, and other physical and chemical properties. For example, ex situ SEI with both inorganic and polymer compounds attracted wide attention because it can combine the merits of these two chemicals. An inorganic and polymer composite ex situ SEI was cast on the lithium foil and successfully operated in lithium symmetrical battery for 200 h even at 10 mA cm^−2^ without the short circuit. The excellent properties were attributed to the hybrid design of flexible polymer (polyvinylene carbonate‐*γ*‐methyl‐propylene trimethoxysilane) and inorganic superionic conductor (Li_7_La_3_Zr_2_O_12_ (LLZO)) (Figure 2b), which promoted the formation of elastic and highly conductive SEI.^[^ ^88^ ^]^

Some recent studies on ex situ SEI are listed in **Table** **2**. As Table 2 shows, compared with the in situ SEI, some ex situ SEIs show limited performance. In these studies, the batteries using ex situ SEI operate stably for no more than 300 cycles, and their CEs are far below 99.9%, which could not meet the requirements of practical application (There is industry lore that for a Li matched full battery, the lifespan should reach 200 cycles and the CE needs to exceed 99.9%^[^ ^93^ ^]^). This is because ex situ films usually cannot form intimate contact with the anode, which leads to the unwanted increment of the internal resistance thus deterioration of the lifespan.^[^ ^94^ ^]^ In order to reduce the contact resistance, an immersing method is developed to obtain ex situ SEI in close contact with lithium. Before assembling the battery, the ex situ SEI forms on the surface of lithium anode by immersing lithium anode in the electrolyte for a period of time. Via simply immersing lithium foil in the mixture of [C_3_mPyr^+^][FSI^−^] and LiAsF_6_, the smooth and compact ex situ SEI formed on the surface of lithium foil at an optimal pretreatment time of 10–12 days.^[^ ^95^ ^]^ The interfacial resistance of pre‐treated lithium anode was no more than 60 Ω cm^−2^, which was close to the resistance of the in situ SEI.^[^ ^96^ ^]^ And the Li//LiFePO_4_ battery prepared by this lithium foil stably operated for 1000 cycles at 1C and the CE of the battery remained 99.89%.^[^ ^95^ ^]^ Even at high current, the ex situ SEI prepared by immersing has obvious improvement on the battery properties. Li and co‐workers^[^ ^90^ ^]^ immersed lithium anode in Mn(NO_3_)_2_‐containing carbonate electrolyte and gained nanotubes array growing on lithium anode (Figure 2c,d). Nanotubes arrays that grew directly on the surface of the lithium anode contained LiF and Li_3_N, apparently enhancing the performance of Li||Cu battery, which stably operated for 150 cycles at high current density of 3 mA cm^−2^ and a CE of 94% was obtained (Although 94% falls far short of the standard of 99.9%, this is a valuable data considering that few studies measure CE at such high current densities). While an inferior performance was presented in the bare Li||Cu battery, with a rapid decay and low CE of 0% after only 85 cycles. This reveals that some good performance (such as high rate) can be achieved by designing the composition and morphology of ex situ SEI. To further meet the requirement of the practical application, significant efforts still need to be put to constantly alleviate the performance of the ex situ SEI.

**Table 2:** Summary of fabrication method and electrochemical performance of recently reported ex situ SEI of lithium anode

Protective layer	Fabrication method	Overpotential for charging/discharging	CE	Ref.
PVCA‐KH570/LLZO	Casting‐evaporation	>100 mV (500 h, symmetric battery, 2 mA cm^−2^, 2 mAh cm^−2^)	<95% (20 cycles, Li||Cu battery, 1 mA cm^−2^, 1 mAh cm^−2^)	[88]
C‐Li_2_S‐LiI layer	Solid–gas reaction	37 mV (>600 h, symmetric battery with C‐Li_2_S‐LiI@Li electrode, 1 mA cm^−2^, –)	Unspecified steady CE (>800 cycles, Li//LTO battery, 1.24 mA cm^−2^, –)	[71]
Zn@Li_2_O	Magnetron sputtering	≈200 mV (1000 h, Li/ZnO@LATP@ZnO/Li symmetric battery, 0.2 mA cm^−2^, –)	–	[97]
Organic/inorganic protective layer	Immersing	20 mV (>500 h, symmetric battery, 5 mA cm^−2^, 1 mAh cm^−2^)	≈94% (>150 cycles, Li||Cu battery, 3 mA cm^−2^, 6 mAh cm^−2^)	[90]
Nafion/LiCl interface	Dip‐casting method	50 mV (1000 h, symmetric battery, 1 mA cm^−2^, 1 mAh cm^−2^)	98.1% (250 cycles, Li||Cu battery, 1 mA cm^−2^, 1 mAh cm^−2^)	[92]
Mineral SEI	Immersing	28 mV (333 cycles, symmetric battery, 1 mA cm^−2^, –)	99.02–99.89% (1000 cycles, Li//LiFePO_4_ battery, 1.25 mA cm^−2^, –)	[95]

Abbreviations: PVCA‐KH570/LLZO, LLZO dispersed in the copolymer of *γ*‐methyl‐propylene trimethoxysilane (KH570) and vinylene carbonate (VC); the constituents of the organic/inorganic protective layer, ROCO_2_Li, Li_3_N, LiF, etc.; major constituents of the mineral SEI, LiF, Li_2_CO_3_, LiSO_2_F, LiOH.

#### 3.1.2 Solid Electrolyte

According to Monroe's report, a solid electrolyte with a high shear modulus (>6.8 GPa) helps suppress lithium dendrites.^[^ ^98^ ^]^ While further study indicated that in addition to the modulus, some other properties, such as Li^+^ conductivity of solid electrolyte, have impact on the effect of suppressing dendrites.^[^ ^37^ ^]^ Moreover, using the solid electrolytes, the drawbacks of commercial liquid electrolytes, such as the leakage and flammability, are circumvented. It's necessary for the solid electrolyte to be designed based on the following characteristics: 1) High Li^+^ conductivity at ambient temperature; 2) reasonable mechanical strength to resist dendrite growth; 3) satisfactory interface compatibility with both electrodes; 4) good chemical and electrochemical stability. Numerous types of solid electrolytes have been investigated, containing ceramic electrolyte, polymer electrolyte, and inorganic/polymer hybrid solid electrolyte.

**Figure 3:**
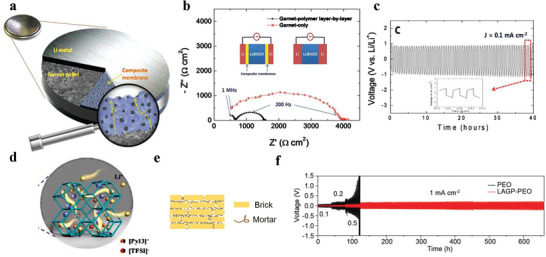
a) Schematic illustration of the layer‐by‐layer design where the polymer membrane acts as an adhesive layer between lithium anode and garnet electrolyte. b) Comparison of Nyquist plots for symmetric batteries with garnet‐polymer layer‐by‐layer and garnet‐only structures, respectively. c) Electrochemical lithium deposition/dissolution cyclic performance of the garnet‐polymer layer‐by‐layer symmetrical batteries at 0.1 mA cm^−2^. a–c) Reproduced with permission.^[^ ^106^ ^]^ Copyright 2019, Wiley‐VCH. d) Schematic illustration of Li^+^ transport paths in IL@MOF electrolyte. Reproduced with permission.^[^ ^110^ ^]^ Copyright 2019, The Royal Society of Chemistry. e) Schematic of “brick‐and‐mortar” microstructure, consisting of LAGP (“brick”) and PEO (“mortar”). f) Cyclic performance of Li||Li symmetric batteries with pure PEO and LAGP‐PEO NCPE. The LAGP‐PEO NCPE improved the lifespan of the symmetric battery apparently. e,f) Reproduced with permission.^[^ ^20^ ^]^ Copyright 2020, Wiley‐VCH.

Inorganic ceramic electrolytes are good conductors of Li^+^ (>10^−3^ S cm^−1^)^[^ ^99^ ^]^ with the high elastic modulus (tens to hundreds of gigapascal).^[^ ^4^ ^]^ Some oxides^[^ ^100^, ^101^, ^102^ ^]^ and sulfides^[^ ^103^, ^104^ ^]^ can be used as electrolytes in lithium metal batteries. However, because of the intrinsic brittleness, rigidity, and poor compatibility of inorganic solid electrolyte/lithium interface, severe challenges appear in the practical battery based on inorganic solid electrolyte. Building an interlayer between the solid electrolyte and lithium can resolve the above problems.^[^ ^105^ ^]^ A membrane consisting of lithium perchlorate, poly(ethylene oxide) (PEO), and garnet particles (**Figure** **3**a) was used as an interlayer between garnet ceramic electrolyte and lithium metal.^[^ ^106^ ^]^ The flexibility of PEO improved the contact between garnet and lithium anode, and the lithium salt enhanced the transport of Li^+^ at the interface. The symmetric battery with this composite membrane displayed four times smaller charge transfer resistance at the interface (≈413 Ω cm^2^) than the battery without membrane (1638 Ω cm^2^, Figure 3b) and stable cycle performance was obtained at 0.1 mA cm^−2^ (Figure 3c), while the garnet‐only battery displayed large polarization (>2000 mV) after operating for only 32 h. Despite this, the interfacial resistances reported in these studies are still too high and thus the current densities applied on the batteries are relatively low.

Polymer solid electrolytes comprise of a blended lithium salts and the polymer matrix.^[^ ^102^ ^]^ Owing to its good flexibility, the interface compatibility of the polymer solid electrolyte is superior to the inorganic solid electrolyte. PEO is the most representative material in polymer solid electrolytes, but like other polymers solid electrolytes, its low modulus (below 400 MPa)^[^ ^107^ ^]^ and insufficient ion conductivity (≈10^−7^ S cm^−1^ at ambient temperature)^[^ ^108^ ^]^ cause severe problems.

Introducing plasticizer (such as, ionic liquid (IL)),^[^ ^108^ ^]^ adding inorganic filler,^[^ ^109^ ^]^ fabricating cross‐linked polymer electrolytes^[^ ^37^ ^]^ are common strategies to enhance the dynamics of Li^+^ transport. Moreover, anchoring anions of the electrolyte salt was also proposed to prompt Li^+^ transport recently. Chen et al.^[^ ^110^ ^]^ prepared ILE@MOF electrolyte, where the metal atom of the MOF (imidazolate framework‐67) strongly interacted with anion TFSI^–^ in electrolyte (Figure 3d) so that Li^+^ moved freely and high ionic conductivity (0.99 × 10^−3^ S cm^−1^ at 30 °C) was obtained. Similarly, Wu et al.^[^ ^111^ ^]^ fabricated Uio‐66 MOF/IL (1‐ethyl‐3‐methylimidazolium bis[(trifluoromethyl)sulfonyl]imide) with high conductivity of 3.2 × 10^−4^ S cm^−1^ at 25 °C. The battery with Uio‐66 MOF/IL showed 380‐cycle retention of 94% at 1C. Actually, the excellent performance of these IL@MOF solid electrolytes also relates to another significant effect induced by anchored anions, and the detailed analyzation will be presented in section 3.2.2. Adding inorganic fillers is another common method to improve both conductivity and mechanical property, which combines the merits of inorganic and polymer solid electrolyte. Hence, the inorganic/polymer hybrid solid electrolyte has been at focus. Numerous polymer matrixes (like PEO,^[^ ^20^, ^112^ ^]^ polyethylene glycol (PEG),^[^ ^113^ ^]^ polycaprolactone,^[^ ^114^ ^]^ etc.) and inorganic fillers (Li_1.5_Al_0.5_Ge_1.5_(PO_4_)_3_ (LAGP),^[^ ^114^ ^]^ Li_10_GeP_2_S_12_ (LGPS),^[^ ^113^, ^115^ ^]^ BN,^[^ ^116^ ^]^ LLZO,^[^ ^102^ ^]^ Ta‐doped Li_7_La_3_Zr_2_O_12_,^[^ ^100^ ^]^ MnO_2_,^[^ ^112^ ^]^ etc.) are mixed to afford good flexibility, conductivity, and rigidity. When the polymer solid electrolyte is combined with inorganic fillers, according to some previous studies, the enhanced conductivity (10^−4^–10^−3^ S cm^−1^)^[^ ^20^, ^102^, ^113^ ^]^ and modulus of the polymer can be one order of magnitude larger than that of the original polymer.^[^ ^20^ ^]^ A LAGP‐PEO hybrid solid electrolyte with “brick‐and‐mortar” microstructure^[^ ^20^ ^]^ (Figure 3e) displayed good stability during cycling (Figure 3f), which can be attributed to its great high Li^+^ conductivity (1.25 × 10^−4^ S cm^−1^ at 25 °C) and flexural modulus (7.8 GPa) provided by the LAGP particles in the PEO matrix. Pan et al.^[^ ^113^ ^]^ prepared the PEO/PEG‐LGPS hybrid solid electrolyte with ultrahigh conductivity (9.83 × 10^−4^ S cm^−1^). While the PEO/PEG electrolyte without LGPS fillers exhibited much lower conductivity of only 1.54 × 10^−4^ S cm^−1^ at room temperature. The polarization of the symmetric battery with LGPS‐PEO/PEG was no more than 80 mV after operating for 6700 h. Liu et al.^[^ ^19^ ^]^ prepared the electrolyte by filling viscoelastic and piezoelectric block‐copolymer electrolytes into a mixed conductor Li_0.33_La_0.56_TiO_3‐_ *_x_* nanofiber film (PPLL). Li_0.33_La_0.56_TiO_3‐_ *_x_* was demonstrated to react with lithium when in contact, generating a hybrid ionic/electron conductive interface (electron conductivity = 0.02 S m^−1^) and minimizing the inhomogeneity of the electric field on the surface of lithium anode. Besides, the piezoelectric effect of block‐copolymer weakened the electronic accumulation at the prominent position on the anodic surface, promoting the smooth deposition of lithium. The capacity retention of LFP/PPLL/Li battery after 550 cycles (0.5 C) was 85%, and the CE was consistently greater than 99.5%. However, the voltage of its control sample dropped sharply at 155 cycles.

**Table** **3** presents the progress of solid electrolyte these years. Most solid electrolytes are still not satisfied with the requirement of high rate, mainly because their limited conductivity are two or three order below that of liquid electrolyte.^[^ ^64^ ^]^ Despite great efforts mentioned above, in solid‐state lithium batteries, uncontrollable growth of dendrites still happens at high current density, leading to the short circuit or capacity fading. The nonuniform contact between solid electrolyte and lithium metal may be related to dendrite formation.^[^ ^117^ ^]^ However, the studies about the mechanism of dendrite formation in solid‐state batteries are still insufficient. For example, Aguesse et al.^[^ ^118^ ^]^ found that lithium clusters appeared in the cavities and pores of the garnet solid electrolyte due to the reduction of Li^+^ by the oxygen backbone in the electrolyte or the garnet electrolyte with nonzero electronic conductivity. So the cracks in the garnet electrolyte would propagate due to the accumulation of lithium metal in the pores until the electrolyte failed. But no continuous dendrite between anode and cathode was observed, and this study had not confirmed whether these clusters were connected with each other via pores. Hence, the detailed mechanism of dendrites formation in the solid electrolyte is still under investigation. Great effort still should be put to address the above issues.

**Table 3:** Summary of physical properties and electrochemical performance of recently reported solid electrolytes used in lithium metal battery

Electrolyte	Conductivity (20–30 °C)	Mechanical property	Overpotential for charging/discharging	Ref.
LAGP‐PEO	1.25 × 10^−4^ S cm^−1^	(flexural modulus) 7.8 GPa	204 mV (500 h, symmetric battery, 60 °C, 1 mA cm^−2^, 1 mAh cm^−2^)	[20]
PEO/PEG‐LGPS	9.83 × 10^−4^ S cm^−1^	—	<80 mV (>6700 h, symmetric battery, 2 mA cm^−2^, 4 mAh cm^−2^)	[113]
LGPS‐PDVC (FG‐SPE)	2.45 × 10^−4^ S cm^−1^	6.67 GPa	≈30 mV (>1200 h, Li/FG‐SPE/Li symmetric battery, 0.1 mA cm^−2^, 0.1 mAh cm^−2^)	[115]
PCL‐SN‐PAN‐LAGP (PSPL)	0.68 × 10^−3^ S cm^−1^	—	16 mV (>500 cycles, Li/PSPL‐16.7%/Li symmetrical battery, 0.1 mA cm^−2^, –)	[114]
ILE (LiTFSI‐[Py13][TFSI])@MOF (ZIF‐67)	0.99 × 10^−3^ S cm^−1^	—	<1600 mV (>1200 h, Li/ILE@MOF/Li battery, 150 °C, 0.5 mA cm^−2^, 1 mAh cm^−2^)	[110]

Abbreviations: PDVC, the copolymer of diallyl carbonate (DAC) and vinylene carbonate (VC); FG‐SPE, functional‐gradient‐structured ultrahigh modulus solid polymer electrolyte; PCL, polycaprolactone; SN, succinonitrile; PAN, poly(acrylonitrile); [Py13][TFSI], *N*‐propyl‐*N*‐methylpyrrolidinium bis(trifluoromethylsulfonyl)imide; ZIF‐67, imidazolate framework‐67.

#### 3.1.3 Insulative Host Material

**Figure 4:**
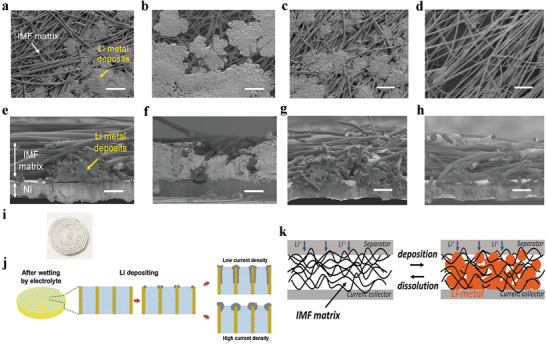
a–d) Top‐view and e–h) side‐view SEM images of the IMF matrixes removed from the batteries after lithium deposition with capacities of a,e) 2.0 and b,f) 10 mAh cm^−2^ and after lithium dissolution with capacities of c,g) 6.0 and d,h) 9.8 mAh cm^−2^. a‐h) Reproduced with permission.^[^ ^120^ ^]^ Copyright 2017, American Chemical Society. i) The photograph of upright lithium anode. j) The schematic illustration of the lithium plating process on upright lithium at the low current density and high current density. i,j) Reproduced with permission.^[^ ^121^ ^]^ Copyright 2019, Wiley‐VCH. k) In the case of insulating IMF matrix, lithium tends to fulfill the internal space of the host. Reproduced with permission.^[^ ^120^ ^]^ Copyright 2017, American Chemical Society.

The enormous volume expansion of lithium has only been extensively studied in recent years. To address this issue, 3D porous materials are introduced into lithium metal battery and serve as host of lithium. Dividing lithium into small domains, batteries using hosts can undertake larger volume variation compared with that using ordinary Li foil.^[^ ^119^ ^]^ Hosts can be classified into two categories: Conductor and insulator. The conductor not only limits volume expansion but regulates the distribution of electrons, which will be introduced in section 3.2.1. Insulative host materials such as polyacrylonitrile (PAN)^[^ ^120^ ^]^ and glass fiber,^[^ ^121^ ^]^ are confirmed effective in physically limiting volume of Li. Using the electrospinning method, PAN insulative microfiber (IMF) was prepared. The deposition of lithium occurred inside the IMF matrix, keeping the thickness of the IMF matrix almost constant (**Figure** **4**a–h) during cycling even at large deposited/stripped lithium of 10 mAh cm^−2^.^[^ ^120^ ^]^ Simultaneously, the insulative host can also inhibit the formation of dendrites. In an example, the direction of dendrite growth was changed by designing the shape of the host. Long‐life lithium metal batteries were obtained by establishing upright Li anodes. The upright structure was constructed by coiling a two‐layer lithium foil/glass fiber into a roll (Figure 4i), which promoted the battery maintain 2500‐cycle capacity of 141 mAh g^−1^ at 1C and 2000‐cycle capacity of 129 mAh g^−1^ at 5C. And the CEs in both cases were almost 100%. The excellent cycle performance is attributed to the upright shape of glass fiber film, where the lithium grows horizontally avoiding the formation of dendrite (Figure 4j).^[^ ^121^ ^]^ Compared with conductive hosts, the insulative hosts avoid lithium only depositing on the top of the three‐dimensional matrix because the Li^+^ tends to gain electrons from the region near the current collector and deposit in the insulative host from bottom to top (Figure 4k). In summary, the insulative host that improves the performance of lithium metal should have the following characteristics: a) Insulative properties, preventing the formation of undesirable lithium metal on the top of the host and effectively using the internal space of the insulative host; b) large internal space, which is advantageous for the storage of deposited lithium metal.^[^ ^120^ ^]^ However, in most strategies, hosts are inactive material. Introducing them probably reduces the energy density of the lithium metal batteries.

### 3.2 Regulating Anode Process

#### 3.2.1 Regulating Electrons/Li^+^ on Anode's Surface

##### Regulating Electrons

Regulating the distribution of electrons on the surface of the anode is vital. Based on Sand's time model:^[^ ^122^ ^]^

(8) $$\tau =\pi D\frac{e^{2}C_{0}^{2}{\left(u_{a}+u_{{\mathrm{Li}}^{+}}\right)}^{2}}{4J^{2}u_{a}^{2}}$$

where *τ* is the Sand's time when dendrites begin to grow, *D* is the diffusion coefficient, *e* is the charge of the electron, *C*_0_ is the initial concentration of lithium salt, *μ*_a_ and *μ*_Li+_ are the mobility of anions and Li^+^ in the electrolyte, and *J* is the effective current density,^[^ ^123^ ^]^ high effective current density *J* contributes to shortening the formation time (*τ*) of dendrites. The uneven distribution of electron on the anodic surface leads to uneven deposition of lithium continuously, which accumulates to form dendrites eventually. The large specific surface area of conductive 3D porous hosts can decrease the effective current density, which is in favor of suppressing the formation of lithium dendrite. And the conductive 3D porous hosts can also serve as a structural framework to hinder volume expansion as insulative hosts mentioned in Section 3.1.3. With these merits, conductive 3D porous host materials gain tremendous momentum in stabilizing lithium anode. Hosts can work as an anodic current collector directly or combine with an ordinary current collector. Before assembling battery, lithium is introduced into the host via infusing molten lithium,^[^ ^124^ ^]^ electroplating,^[^ ^21^ ^]^ or mechanically pressing.^[^ ^125^ ^]^

**Figure 5:**
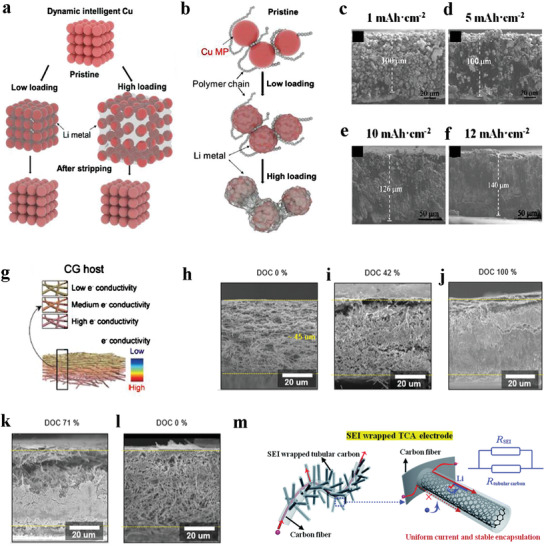
a) Dynamical changes of DICu current collector in lithium deposition/dissolution. b) The working mechanism of the DICu at low/high loading of lithium. When the loading is relatively low, the association among Cu MP (microparticles) depends on the interaction between Cu MP and polymer chain, while lithium becomes “binder” instead of PVDF when the loading of lithium increases. c–f) SEM images of DICu current collectors after plating lithium with different deposition capacities. a–f) Reproduced with permission.^[^ ^129^ ^]^ Copyright 2020, American Chemical Society. g) Schematic structure of the CG (conductivity gradient) host. h–l) The cross‐sectional side‐view ex situ SEM images of CG electrode at different DOC (depth of charge) indicated the fully utilize of internal space during a whole plating/stripping process. (DOC 0%, 42%, 100%, 71% represented that the lithium loading in the CG host were 0 mAh cm^−2^, 3 mAh cm^−2^, 7 mAh cm^−2^ (fully lithiated/delithiated state), and 2 mAh cm^−2^. g–l) Reproduced with permission.^[^ ^22^ ^]^ Copyright 2020, Wiley‐VCH. m) Schematic illustration of electron‐transport pathway and Li plating in the SEI wrapped TCA electrode. Reproduced with permission.^[^ ^132^ ^]^ Copyright 2019, The Royal Society of Chemistry.

Various carbon materials (multilayer graphene,^[^ ^126^ ^]^ carbon nanotubes,^[^ ^124^ ^]^ 3D carbon fiber,^[^ ^127^ ^]^ etc.), copper‐based material (interconnected nanoparticle,^[^ ^128^ ^]^ microparticle,^[^ ^129^ ^]^ fiber,^[^ ^130^ ^]^ etc) and stainless steel mesh^[^ ^131^ ^]^ have been confirmed effective host in previous studies. For example, Chen et al.^[^ ^129^ ^]^ designed a dynamic intelligent Cu (DICu) current collector composed of Cu microparticles and polymer (severe as “binder”) (**Figure** **5**a,b). Due to the low current density induced by the large specific surface area of DICu, Li||DICu battery exhibited long‐term stability with a steady overpotential for charging/discharging (≈879.5 mV, 1000 h) even at 10 mA cm^–2^, while the battery using planar Cu as current collector failed in less than 100 h. Significantly, DICu can store a large amount of lithium (Figure 5c–f). It is due to its tunable distance between Cu particles, adapting to larger volume expansion automatically.

However, owing to the shorter diffusion length of Li^+^ from the electrolyte to the upper surface than to the lower surface, lithium preferentially deposits on the upper surface of these hosts, causing inadequate use of 3D space. The problem is more pronounced at large current density. To solve this problem, some hosts with uneven electronic conductivity have been designed to get desired deposition of lithium. A heterofibrous host^[^ ^22^ ^]^ (Figure 5g) with gradient electrical conductivity was fabricated. The conductivity gradient host consisted of three layers of material with gradually varied conductivity. Because electrons were more readily available in highly conductive regions, deposition occurred preferentially at the bottom layer and the internal space of host was fully utilized during plating/stripping of lithium (Figure 5h–l). In another example, an SEI wrapped tubular carbon array (TCA) growing on long carbon fibers (Figure 5m) was proposed, and the electron‐insulated SEI layer at the outer surface of the TCA created higher impedance than the inner surface. This resulted in selective electron transport along with the tubular carbon to/from the current collector, rather than through the SEI layer. So the deposition of lithium occurred inside the ordered tubular carbon. In this way, the Li||SEI wrapped TCA battery performed a good durability (overpotential for charging/discharging was no more than 60 mV after 800 h) under a relatively high load of 2 mAh cm^−2^.^[^ ^132^ ^]^

##### Regulating Both Electrons and Li^+^

**Figure 6:**
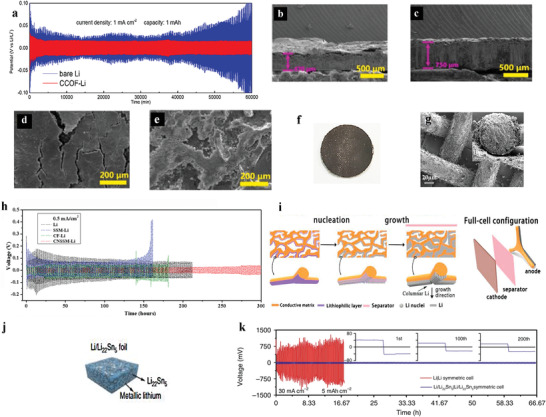
a) A comparison of the stability of CCOF‐Li and bare lithium anode in symmetrical batteries. The side‐view SEM images of b) the bare lithium anode and c) the CCOF‐Li electrode after 100 cycles. The top‐view SEM images of d) the bare lithium anode and e) the CCOF‐Li electrode after 100 cycles. The bare lithium anode has a markedly thicker white dead‐lithium layer. The original thickness of bare‐lithium electrode and CCOF‐Li electrode are 380 and 750 µm, respectively, indicating a larger volume expansion of bare‐lithium anode. a–e) Reproduced with permission.^[^ ^140^ ^]^ Copyright 2019, Elsevier. f) Photograph and g) SEM images of CNSSM‐Li composite electrode. h) Comparison of cyclic performance for symmetrical Li||Li batteries using pristine Li foil, stainless steel mesh (SSM)‐Li, carbon nanofibers (CF)‐Li, or carbon‐nitrogen modified stainless steel mesh (CNSSM)‐Li electrodes at the current density of 0.5 mA cm^−2^. f–h) Reproduced with permission.^[^ ^125^ ^]^ Copyright 2020, Elsevier. i) Illustration of the lithium nucleation and growth process on the backside of the host, in which the growth direction of lithium can be guided. Reproduced with permission.^[^ ^135^ ^]^ Copyright 2019, Elsevier. j) The schematic illustration of mixed electron/Li^+^ conductive electrode composing of Li_22_Sn_5_ and lithium. k) The comparison of stability between Li||Li symmetric battery (red) and Li/Li_22_Sn_5_||Li/Li_22_Sn_5_ symmetric battery (blue). j,k) Reproduced with permission.^[^ ^142^ ^]^ Copyright 2020, Springer Nature.

Many ordinary hosts, like some carbon‐based and copper‐based materials, are lithiophobic. The low affinity between these materials and lithium leads to uneven flux of lithium ions and generally brings about inhomogeneous lithium nucleation and deposition.^[^ ^133^ ^]^ In some studies, the distribution of Li^+^ on the anode surface is regulated and a more uniform deposition of lithium is achieved by simultaneously adjusting electrons and Li^+^ on the anode surface. Uniformly decorating hosts using lithiophilic materials with low overpotential for Li nucleation is a common strategy.^[^ ^134^ ^]^ Some metals (Au,^[^ ^135^ ^]^ Ag,^[^ ^136^, ^137^ ^]^ Zn, Sn^[^ ^134^ ^]^), metallic oxides (TiO_2,_^[^ ^138^ ^]^ Co_3_O_4,_^[^ ^139^ ^]^ Cu_2_O,^[^ ^140^ ^]^ ZnO^[^ ^23^ ^]^), and other materials (carbon with defects^[^ ^141^ ^]^ or heteroatom,^[^ ^125^ ^]^ porphyrin^[^ ^119^ ^]^) are decorated on 3D current collectors as lithiophilic sites. Li^+^ is guided by these lithiophilic sites to deposit uniformly inside the host when batteries are charged. In one example, cuprite coated Cu foam host (CCOF‐Li) electrode^[^ ^140^ ^]^ showed smaller overpotential (15.8 mV at 1 mA cm^−2^) in the test of stability in symmetric batteries than bare lithium (>200 mV, **Figure** **6**a). And negligible dead‐lithium layer and volume expansion (Figure 6b,c) of the CCOF‐Li electrode were also observed using SEM. Owing to the existence of these lithiophilic sites, a more uniform lithium configuration was observed in CCOF‐Li electrode's SEM image after 100 cycles, while that of bare‐Li electrode displayed many cracks on the surface of lithium anode (Figure 6d,e). He et al.^[^ ^125^ ^]^ coated lithiophilic graphitic carbon nitride (g‐C_3_N_4_) on stainless steel mesh (Figure 6f,g) and gained better stability compared with stainless steel mesh without lithiophilic sites (Figure 6h). Fu et al.^[^ ^21^ ^]^ treated the copper nanowires with sodium formate dihydrate (SF‐CUNW) to reconstruct copper nanowires’ surface into a (110) dominated surface. As a result, the lithiophilic properties of copper nanowires were greatly improved. Moreover, it was found that a passivation layer formed on SF‐treated surface, which inhibited the surface oxidation of CUNW. It was the first time that the property's degradation caused by Cu‐based host materials’ oxidation was reported. With low N/P ratio of only 1.6:1, lithiophilic and antioxidant Li/SF‐CuNW//LFP battery retained a high CE of 99.8% at 1.87 mA cm^−2^ after 1000 cycles. While the capacity of Li foil//LFP battery exhibited a sharp decline after 100 cycles. Considering that dendrites cannot be fully eliminated due to the inherent thermodynamic tendency, Hou et al.^[^ ^135^ ^]^ altered the growth direction of lithium by sputtering Au on copper foam's backside (Figure 6i). The Li^+^ were guided to the copper foam's backside and deposited, which reduced the risk of the separator being destroyed and obtained enhanced stability of lithium metal battery. The symmetrical battery using the Au modified copper foam operated stably for 15 000 min, while the battery using bare copper foam was short‐circuit after 3500 min.

Besides lithiophlic material, some other methods were put forward to regulate both electrons and Li^+^, which were proved to be useful. Wan et al.^[^ ^142^ ^]^ reported a mixed electron and lithium‐ion conductive Li_22_Sn_5_ network (Figure 6j). Even under 30 mA cm^−2^ and with areal capacity of 5 mAh cm^−2^, excellent stability (overpotential for charging/discharging = 20 mV, after 200 cycles) was obtained for the Li/Li_22_Sn_5_||Li/Li_22_Sn_5_ battery (Figure 6k). Good performance was attributed to the uniform distribution of both Li^+^ and electrons in the electron/Li^+^ conductive network. Considering that introducing inactive host material reduces the energy density of the battery, direct synthesis of lithium metal with 3D morphology is effective to avoid this disadvantage. Wang et al.^[^ ^143^ ^]^ prepared thiophdiyne (TD) membranes on copper foil, in which the thiophene groups were lithiophilic. By adjusting the distribution of Li^+^ during the battery's operation, seaweed‐like lithium was eventually formed on the lithiophilic copper foil decorated by TD (TD‐SW‐Li). Seaweed‐like lithium effectively reduced the current density, which further suppresses dendrites’ growth. The Cu─Li//LTO battery decayed rapidly after 400 cycles at 1C and 100 cycles at 5C. However, the TD–SW–Li//LTO battery showed stable capacity retention for more than 1000 cycles at both 1 and 5C owing to its stable lithium anode.

**Figure 7:**
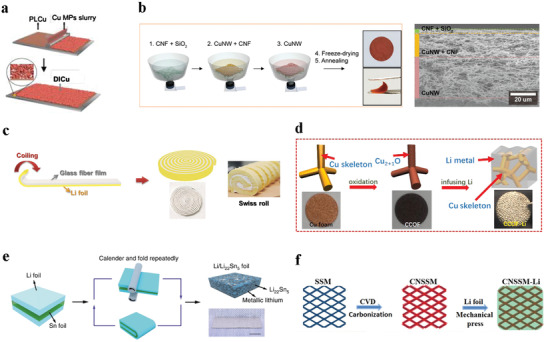
a) The fabrication of DICu current collectors with granular piling structure by solution‐casting commercial Cu micro‐particles (MPs) onto Cu foils. Reproduced with permission.^[^ ^129^ ^]^ Copyright 2020, American Chemical Society. b) The fabrication of the CG host via vacuum‐assisted infiltration process. Reproduced with permission.^[^ ^22^ ^]^ Copyright 2020, Wiley‐VCH. c) The preparation of the CLi by coiling glass fiber film and Li foil into the vortex‐shape structure, like a Swiss roll. Reproduced with permission.^[^ ^121^ ^]^ Copyright 2019, Wiley‐VCH. d) The two‐step method to fabricate the CCOF‐Li electrode. Reproduced with permission.^[^ ^140^ ^]^ Copyright 2019, Elsevier. e) The preparation of the Li/Li_22_Sn_5_ nanocomposite foil realized by a facile calendaring and folding route. Reproduced with permission.^[^ ^142^ ^]^ Copyright 2020, Springer Nature. f) The fabrication process of the CNSSM, final carbon‐nitrogen modified stainless steel mesh, and lithium composite electrode (CNSSM‐Li). The SSM was first carbonized and covered with a dense functional layer, then pressed onto lithium foil via mechanical pressing. Reproduced with permission.^[^ ^125^ ^]^ Copyright 2020, Elsevier.

To analyze the progress of hosts, some recent reports are summarized in **Table** **4**. From Table 4, most hosts can bear relatively large current density because of their large specific surface area. But the coulombic efficiency of them cannot meet the requirement of practical application (>99.9%).^[^ ^93^ ^]^ According to the calculation about dendritic growth based on the phase field model, the quantity of SEI formed in the battery with 3D host is proportionally to the increasing electroplated capacity of lithium, and has no significant relationship with the basic properties of 3D hosts (such as, channel size).^[^ ^144^ ^]^ Hence, the host material is not as effective as in situ/ex situ SEI or the solid electrolytes in reducing SEI formation and thus improving CE. The fabrication processes of some hosts are also summarized (Table 4, **Figure** **7**). Some methods, such as, coiling and solution casting, are facile and low energy consumed. These processes are beneficial to large‐scale production of lithium metal batteries.

**Table 4:** Summary about the fabrication and performance of some hosts used in lithium metal batteries

Host material	Fabrication	Overpotential for charging/discharging	CE	Ref.
DICu	Solution casting	≈879.5 mV (1000 h, Li/DICu||Li/DICu battery, 10 mA cm^−2^, 1 mAh cm^−2^)	99.6% (800 cycles, DICu||Li/DICu battery, 1 mA cm^−2^, 1 mAh cm^−2^)	[129]
CG host (CNF@SiO_2_/ CuNW@CNF/ CuNW)	Vacuum‐assisted infiltration	<100 mV (100 cycles, Li||Li@host asymmetric battery, 5 mA cm^−2^, 1 mAh cm^−2^)	>96% (120 cycles, Li||Li@host asymmetric battery, 0.5 mA cm^−2^, –)	[22]
SEI wrapped TCA	Hydrothermal method/template method/charge–discharge	<60 mV (800 h, Li||SEI wrapped TCA battery, 5 mA cm^−2^, 2 mAh cm^−2^)	>98% (200 cycles, Li|| SEI wrapped TCA battery, 1 mA cm^−2^, 2 mAh cm^−2^)	[132]
CNSSM	Carbonization/chemical vapor deposition	<100 mV (300 h, symmetric battery, 0.5 mA cm^−2^, –)	—	[125]
CCOF	Annealing in air	15.8 mV (60000 min, CCOF‐Li symmetric battery, 1 mA cm^−2^, 1 mAh cm^−2^)	—	[140]
Au modified Cu foam	Magnetron sputtering	≈50 mV (15000 min, symmetric battery, 0.5 mA cm^−2^, 0.5 mAh cm^−2^)	>98.1% (163 cycles, LiǁCuF@Au battery, 1 mA cm^−2^, 1 mAh cm^−2^)	[135]
Li/Li_22_Sn_5_ nanocomposite	Calendaring and folding	10 mV (200 h, Li/Li_22_Sn_5_||Li/Li_22_Sn_5_ symmetric battery, 10 mA cm^−2^, 5 mAh cm^−2^)	—	[142]

Abbreviations: DICu, dynamic intelligent Cu; CG, conductivity gradient; CNF, cellulose nanofibers; CuNW, copper nanowires; TCA, tubular carbon array; CNSSM, g‐C_3_N_4_ modified stainless steel mesh; CCOF, cuprite‐coated Cu foam.

#### 3.2.2 Adjust Li^+^ in Electrolyte

The mass transport in the electrolyte is one of the most important steps in the process of lithium deposition. Adjusting the distribution of Li^+^ in the electrolyte near anode can affect the deposition of lithium. Several solutions are proposed to regulate Li^+^ in the electrolyte.

##### Adjust the Li^+^ Flux

**Figure 8:**
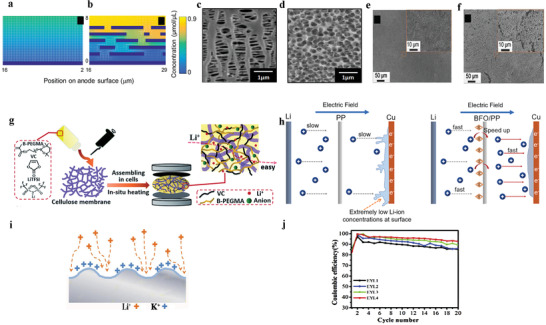
Simulation of Li^+^ distribution in the electrolyte for the battery a) without and b) with porous membrane. a,b) Reproduced with permission.^[^ ^145^ ^]^ Copyright 2018, American Chemical Society. SEM of the pore structure for c) the PP membrane and d) the 3DOM PI membrane. c,d) Reproduced with permission.^[^ ^146^ ^]^ Copyright 2019, American Chemical Society. Postmortem SEM images of the lithium symmetric battery electrodes obtained after cycling with e) CPC and f) PE separators. e,f) Reproduced with permission.^[^ ^147^ ^]^ Copyright 2018, Wiley‐VCH. g) Schematic illustration of fabricating P(V‐B) solid electrolyte and the effect of immobilizing anions. Reproduced with permission.^[^ ^151^ ^]^ Copyright 2019, The Royal Society of Chemistry. h) Schematic diagram of the ferroelectric effect of BiFeO_3_ promoting the transmission of lithium ions. Reproduced with permission.^[^ ^152^ ^]^ Copyright 2021, Elsevier. i) Self‐healing electrostatic shielding effect. K^+^ additive gathered around initial growth tip of lithium metal dendrite. The electrostatic force between K^+^ and Li^+^ causes lithium to deposit in the pits. j) The cyclic performance of the Cu//NMC batteries with the electrolytes when operated at 0.2 mA cm^−2^. (EYL1 is the baseline electrolyte consisting of ethylene carbonate (EC) and diethyl carbonate. EYL2 is the electrolyte introducing KPF_6_ additive in the EYL1. EYL3 is the electrolyte introducing TMSP (tris (trimethylsilyl) phosphite, the function of TMSP is protecting cathode, which isn't the key point we focus on) additive in the EYL1. EYL4 is the electrolyte introducing KPF_6_ additive in the EYL3. i,j) Reproduced with permission.^[^ ^153^ ^]^ Copyright 2019, Elsevier.

In the bulk phase of the electrolyte, when the effects of migration and convection on solute dispersion are neglected, Li^+^ concentration linearly distributes in the *y*‐direction and without variations in the *x*‐direction (**Figure** **8**a). This concentration gradient leads to the proliferation of dendrites because the Li^+^ concentration around the protruding portion of the anodic surface is higher than that around other regions of anodic surface. According to Li and co‐workers’ simulation,^[^ ^145^ ^]^ porous structure could generate movement of Li^+^ in the *x*‐direction. The phenomenon was owing to the diffusion difficulty of Li^+^ in tortuous structure (Figure 8b). In the real electrolyte, the *x*‐direction expands into a two‐dimensional plane. Therefore, from micro perspective, porous media facilitates the transport of lithium ions among the pores, promoting homogeneous distribution of Li^+^ and benefiting for smooth deposition of lithium metal.

The separator with ordered porous structure is conducive for the Li^+^ flux in the electrolyte to pass through the separator uniformly. An ultrafine porous polyimide (PI) separator^[^ ^146^ ^]^ with a 3D ordered macroporous structure (Figure 8c,d) displayed better stability compared with ordinary polypropylene (PP) separator with random porous structure. In another example, nanocellulose modified polyethylene separators (cellulose nanofibers/polyethylene/cellulose nanofibers, referred to as CPC)^[^ ^147^ ^]^ generated uniform Li^+^ flux and led to smooth deposition (Figure 8e,f). In contrast to ordinary PE separator, the homogenous nanochannels of CPC contribute to adjusting the Li^+^ flux. The overpotential of the lithium symmetric battery with CPC separator was no more than 400 mV and remained almost constant for 155 cycles at the current density of 0.65 mA cm^−2^. However, the battery using ordinary PE separator performed much higher overpotential (>1000 mV) in the same conditions. Other similar studies, such as three‐dimensionally ordered macroporous polybenzimidazole separator,^[^ ^148^ ^]^ sandwich‐structured separator composed of two cellulose nanofiber (CNF) surface layers and an intermediate glass microfiber and CNF composite layer,^[^ ^149^ ^]^ were demonstrated effective in prompting lithium deposition uniform, indicating the significance of adjusting flux of Li^+^. The artificial SEI with uniform pores serves a similar purpose. Zhang et al.^[^ ^150^ ^]^ sprayed microporous thermo‐setting polymer (CMP) on the surface of lithium metal. The negatively charged nanofluid channel on the CMP enabled rapid selective transmission of Li^+^, promoting the uniform distribution of Li^+^ and smooth deposition of lithium. In addition, CMP layer also prevented the lithium from side reaction. The as‐assembled CMP‐Li//NCM811 pouch battery with low N/P ratio (1.34) and lean electrolyte (2.5 g Ah^−1^) achieved energy density exceeding 400 Wh kg^−1^ and cycle lifespan >65 cycles.

Some solid electrolytes and separators are also given the ability to regulate the movement of lithium ions by immobilizing anion of lithium salts. As an example showed Ma et al.^[^ ^151^ ^]^ synthesized solid electrolyte by polymerizing poly(ethylene glycol) methyl ether methacrylate containing cyclic boroxane groups and vinylene carbonate on a cellulose membrane framework (referred to as P(V‐B)). The boron moieties in this solid electrolyte contributed to immobilizing anions (Figure 8g), thereby made the distribution of Li^+^ more uniform and led to smoother lithium plating than ordinary electrolyte. The battery with P(V‐B) exhibited good stability even at 4C after 600 cycles. Some ceramics, such as, Li_3_ *_x_*La_2/3−_ *_x_*TiO_3_^[^ ^107^ ^]^ and LAGP,^[^ ^114^ ^]^ also have anchoring effect on anions and display single‐ion conductivity, which are also demonstrated effective in promoting uniform distribution of Li^+^ in the electrolyte. The physical properties of ferroelectric materials are also used to regulate the transport of lithium ions. Xue et al.^[^ ^152^ ^]^ prepared a BiFeO_3_/PP composite membrane and used it in a pouch lithium‐sulfur battery. The pouch batteries exhibited a stable cyclic performance at 100 mA/pouch battery for 150 cycles. For comparison, a dramatic fluctuation of CE was observed just after 15 cycles for the pouch battery with ordinary PP separator. The better cycling performance of pouch battery was due to the polarization of BiFeO_3_ under the external electric field. This ferroelectric effect reduced the diffusion energy barrier of Li^+^, thus reducing the Li^+^ depletion nearby the surface of the lithium anode and obtaining a smoother lithium deposition (Figure 8h).

##### Self‐Healing Electrostatic Shielding Effect

Self‐healing electrostatic shielding (SHES) effect is also a direction to adjust Li^+^ in the electrolyte. Additives like K^+^,^[^ ^153^ ^]^ Cs^+^,^[^ ^154^ ^]^ Rb^+[^ ^155^ ^]^ are added in electrolyte, and when the concentration of these cations is under a certain amount, their reduction potential will be lower than that of Li^+^ in electrolyte. Therefore, they do not deposit at anode before Li^+^ does. After lithium plating, the additive cations distribute around the tip of early dendrites and produce electrostatic shield against positive charges (Figure 8i). The effect prompts further deposition of Li^+^ to regions adjacent to early dendrites, suppressing the growth of dendrites.^[^ ^154^ ^]^ This effect of inhibiting dendrites was viewed by Li and co‐workers using in situ optical microscopy. At the current of 0.1 mA, after depositing for 1500 s, many protrusions appeared on the anodic surface of the transparent quartz battery with normal electrolyte (LiPF_6_‐ethylene carbonate (EC)/dimethyl carbonate (DMC)), while the anodic surface of the battery with Rb^+^ added electrolyte was smooth.^[^ ^155^ ^]^ However, although the SHES effect improves the morphology of lithium anode during lithium deposition, the CE of the lithium metal battery is still relatively low. A LiPF_6_‐EC/DEC electrolyte with KPF_6_ and TMSP (tris (trimethylsilyl) phosphite) as additives made Cu//NMC battery show a little improvement of CE compared with the battery without additives (Figure 8j).^[^ ^153^ ^]^ The average CE of Cu//NMC battery with KNO_3_ additive for 41 cycles is only 95.21% at the current density of 1 mA cm^−2^.^[^ ^156^ ^]^ The limited electrochemical performances of this strategy possibly result from that no effective SEI formed on the surface of lithium anode, which cannot prevent lithium metal from being corroded by electrolyte components.^[^ ^154^ ^]^ It is necessary to further study the fundamental mechanism in this field and find effective solution to enhance the modification effect of this strategy on lithium anode.

#### 3.2.3 Regulate the Free Solvent Molecules

**Figure 9:**
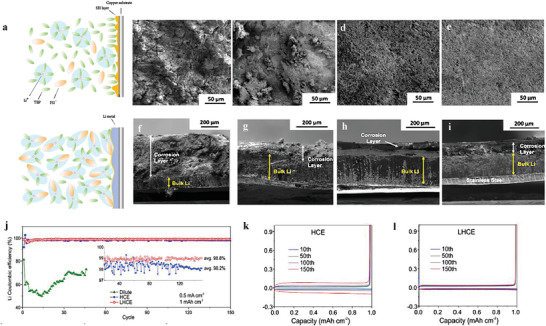
a) Schematic illustrations of the lithium metal plating on a Cu collector in dilute electrolyte (up) and HCE (down). Reducing the amount of free solvent (TEP, triethylphosphate) is in favor of reducing the decomposition of solvent molecules on the surface of lithium anode. Reproduced with permission.^[^ ^158^ ^]^ Copyright 2019, American Chemical Society. b–e) Top and f–i) side‐view SEM images of lithium after cycling using b,f) E‐control (the traditional electrolyte consisting of 1.0 m LiPF_6_ in EC/EMC with VC as additive) and three LHCEs: c,g) LiFSI in DMC‐BTFE (bis(2,2,2‐trifluoroethyl) ether), d,h) LiFSI in EC/EMC‐BTFE, and e,i) LiFSI in EC/EMC‐BTFE with lithium difluoro(oxalate)borate (LiDFOB) as an additive. From the results, LHCEs are effective to attenuate the thickness of the corrosion layer on lithium surface. b‐i) Reproduced with permission.^[^ ^163^ ^]^ Copyright 2018, American Chemical Society. j) The CEs with cycling in different electrolytes measured in Li||Cu batteries. k,l) Voltage profiles of Li plating and stripping processes at selected cycles with k) HCE (LiFSI‐TMS (tetramethylene sulfone)), and l) LHCE (LiFSI‐TMS‐TTE) as electrolytes. j–l) Reproduced with permission.^[^ ^160^ ^]^ Copyright 2018, Elsevier.

Because of the strong reducibility of lithium, lithium is highly reactive toward most non‐aqueous electrolytes.^[^ ^157^ ^]^ According to Xiao's report,^[^ ^158^ ^]^ the reduction potential of solvent fitted Nernst equation, which shifted negatively with the increasing concentration of the electrolyte. As a result, the reactive activity of the solvent decreased. In this study, a mechanism was also proposed to explain why the HCE reduced the reactivity of solvent molecules (**Figure** **9**a). Li^+^ in ordinary electrolyte are all coordinated with the solvent molecules to form solvate around Li^+^, and there are plenty of free solvent molecules that are readily reactive with lithium. While in HCE (the molar ratio of salt to solvent ≥1:2), the majority of solvent molecules are coordinated with Li^+^, inducing largely improved reduction stability of the solvent, reducing the corrosion of lithium metal. Highly concentrated fluorine‐organic Li salt electrolyte has been used to prevent the corrosion of lithium in previous reports. For example, Dong et al.^[^ ^159^ ^]^ characterized the reactive activity of lithium metal toward 1 m LiTFSI‐TEP (triethyl phosphate) electrolyte and 2.8 m LiTFSI‐TEP electrolyte by soaking lithium foil into the above two electrolytes. The results demonstrated that lithium gradually dissolved in the dilute electrolyte, but lithium metal's surface only turned dim a little in the 2.8 m solution, indicating greater stability of lithium metal in HCE. In long‐term test of the lithium symmetric batteries with these two electrolytes, the battery based on 1 m LiTFSI‐TEP electrolyte operated steadily for less than 150 h, ending with a fast polarization increase of the voltage. In contrast, the battery based on 2.8 m LiTFSI‐TEP electrolyte performed very stable cyclic performance for 250 h, which could be attributed to the reduced side reactions between lithium and solvent. Nevertheless, the high salt concentration results in high cost, high viscosity, and poor electrolyte‐electrode wettability, which greatly hinder its practical application. Recently, researchers proposed the concept of “locally concentrated electrolytes (LHCE).” LHCE is a mixture of concentrated electrolyte and inactive diluent (a kind of liquid that is inert to lithium). Thus, it not only overcomes the disadvantage of HCE mentioned above, but also remains high salt/solvent molecule ratio to prevent the corrosion of lithium (Figure 9b–i). According to Ren's study,^[^ ^160^ ^]^ sulfones’ viscosity and wettability issues were overcome by adding fluorinated ether, 1,1,2,2‐tetrafluoroethyl‐2,2,3,3‐tetrafluoropropyl ether (TTE, diluter) in the 1.2 m LiFSI‐tetramethylene sulfone (TMS) electrolyte. This LHCE displayed low viscosity of only 14.1 cP at 25 °C, in contrast to the 99.5 cP of corresponding HCE (same electrolyte formula but without TTE). Simultaneously, the conductivity of the electrolyte increased from 1.74 to 2.03 mS cm^−1^ at 25 °C. The enhanced properties of LHCE induced higher CE and less polarization of the Li||Cu battery (0.5 mA cm^−2^, 150 cycles), in contrast to the unstable profiles of Li||Cu battery using HCE (Figure 9j–l). The electrochemical performance of Li||Cu battery using HCE and LHCE are summarized in **Table** **5**. Although HCE and LHCE has been confirmed to be effective in modifying lithium anode, however, according to the Table 5, the rate performance and the coulombic efficiency of lithium metal batteries still cannot meet the requirement of practical application.^[^ ^93^, ^161^ ^]^ The concentrated dual‐salt system has been confirmed effective for improving ionic conductivity. Generally, the salts with low interaction energy between cations and anions are more likely to dissociate and form free ions, which results in high ionic conductivity. On the contrary, the salts with high interaction energy will produce a large number of cation‐anion related complexes, which can form a stable SEI through preferentially being reduced on the surface of anode. Therefore, the mixture of salts with different properties can get better modification effect. For example, Pham et al.^[^ ^24^ ^]^ chose lithium difluorophosphate (LiDFP)‐LiTFSI that had the highest and lowest interaction energy, respectively. 2.2 m LiDFP and 1.23 m LiTFSI in 1,2‐dimethoxyethane were proposed as a promising electrolyte system due to its high conductivity (6.57 mS cm^−1^), which was higher than the conductivity of the control groups with LiDFP or LiTFSI alone. Using the optimal electrolyte system, the potential of the symmetrical lithium metal batteries remained constant for over 400 h (1600 cycles) under high current density of 8 mA cm^−2^.

**Table 5:** Summary of the electrochemical performance for recently reported HCE and LHCE used in lithium metal batteries

Electrolyte	Type	CE	Ref.
7 m LiFSI in FEC	HCE	99.64% (400 cycles, Li||Cu battery, 0.25 mA cm^−2^, –)	[93]
2.8 m LiTFSI in TEP	HCE	90.8% (80 cycles, Li||Cu battery, 0.2 mA cm^−2^, 0.2 mAh cm^−2^)	[159]
1.2 m LiFSI in EC/EMC + BTFE (diluter) + 0.15 m LiDFOB (additive)	LHCE	≈98.5% (200 cycles, Li||Cu battery, 0.5 mA cm^−2^, 1 mAh cm^−2^)	[163]
3.2 m LiFSI in TEP + BTFE (diluter) (salt's concentration ≈1 m)	LHCE	99.3% (350 cycles, Li||Cu battery, 0.2 mA cm^−2^, 1 mAh cm^−2^)	[158]
LiFSI in TMS + TTE (diluter) (molar ratio = 1:3:3)	LHCE	98.8% (150 cycles, Li||Cu battery, 0.5 mA cm^−2^, 1 mAh cm^−2^)	[160]

The solvation sheath of Li^+^ also has an effect on the low‐temperature performance of lithium metal batteries. Holoubek et al.^[^ ^162^ ^]^ investigated the low temperature performance of lithium metal batteries with low N/P capacity ratio. The quantum chemical simulation of bond energies of Li^+^(solvent)*_n_* complexes in different solvent systems at low temperature was carried out, and yielded binding energies of −414 and −280 kJ mol^−1^ for the Li^+^(DME)_2.3_ (1,2‐dimethoxyethane) and Li^+^(DEE)_1.8_ (diethyl ether) complexes, respectively. Strong bond energy led to higher charge transfer resistance, promoting the uneven deposition of lithium at low temperature. A high‐loading 3.5 mAh cm^−2^ sulfurized polyacrylonitrile cathode was paired with excess lithium anode. When cycling at −40 and −60 °C, respectively, the battery with DEE electrolyte system retained 84% and 76% of its room temperature capacity, showing stable performance during 50 cycles and outperforming the 38.9% and 2.8% of battery with DME electrolyte system.

### 3.3 Other Strategies

**Figure 10:**
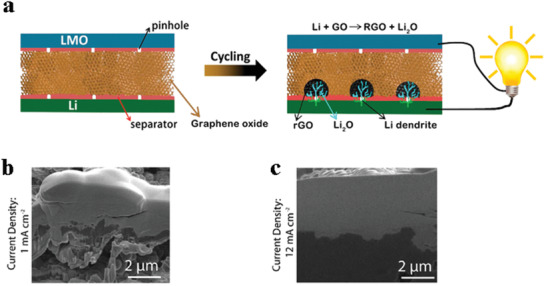
a) Schematic of tri‐layer separator suppressing Li dendrites in LMO/GO/Li battery. The middle‐layer GOF etches dendrites to hinder their propagation. Reproduced with permission.^[^ ^164^ ^]^ Copyright 2018, Elsevier. Cross‐sectional images of lithium metal electrode in the Li||Li symmetric battery that operated at b) ≈1 mA cm^−2^ and c) ≈12 mA cm^−2^. b,c) Reproduced with permission.^[^ ^165^ ^]^ Copyright 2019, Elsevier.

In addition to the two methods mentioned above, there are some other methods to stabilize lithium anode. In a tri‐layer separator, oxidized graphene film layer sandwiched by two pieces of polypropylene (PP) reacted with dendrite spontaneously when the dendrite pierced one of the PP layers (**Figure** **10**a). The lithium metal battery containing this separator ran for 6000 cycles at 2C without short circuit.^[^ ^164^ ^]^ The proper charging protocol (>10 mA cm^−2^) of the battery made dendrites heal at high current density (Figure 10b,c), which was attributed to the enhanced self‐diffusion of lithium atoms at high temperature arising from the high current density.^[^ ^165^ ^]^ Furthermore, optimizing assembling process can also play a role in modifying the lithium anode, such as using a small‐area Cu electrode in Li||Cu battery.^[^ ^44^ ^]^ These methods sometimes exhibit excellent performance. Therefore, to get more certain evidence, extensive research is needed.

The literature reviewed above is the strategies to modify general issues of lithium anodes in lithium metal batteries. Nevertheless, in Li‐O_2_ batteries, lithium anodes confront other challenges. To ensure the energy density of Li‐O_2_ batteries, in the practical Li‐O_2_ batteries system, oxygen is directly obtained from the air. H_2_O and CO_2_ in the air will cause insulative by‐products (such as, LiOH and Li_2_CO_3_) on lithium anodes, blocking the electrode reaction and greatly reducing the cycling performance of the battery.^[^ ^5^ ^]^ Such harsh working conditions place higher requirements on the quality of SEI. More robust and chemically stable SEI needs to be studied to improve the environmental stability of lithium anodes, which may be one of the future research directions of practical lithium metal batteries. Li‐O_2_ battery is a field that should be explored in depth. Since this review focuses on the general issues of lithium anodes, the detailed analysis about Li‐O_2_ batteries is not included.

## 4 Advanced Simulation and Calculation

**Figure 11:**
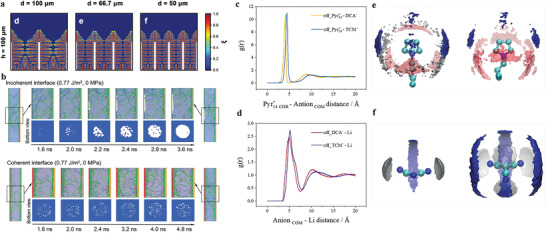
a) The simulation of dendrites’ morphology formed in 3D conductive current collector with channel length of 100 µm and different channel widths. Reproduced with permission.^[^ ^144^ ^]^ Copyright 2019, Elsevier. b) Lithium deposits on the incoherent interface and coherent interface, respectively. When lithium is in contact with the solid state electrolyte, the pores formed on the interface can be seen in the side view (upper) and the bottom view (lower). Reproduced with permission.^[^ ^25^ ^]^ Copyright 2021, Wiley‐VCH. The analysis of solvation sheath via the MD simulations. c) The radial distribution function of the Pyr_14_^+^ COR and DCA−COM or TCM−COM. d) The radial distribution function of lithium ion and DCA−COM or TCM−COM. e) Refer to the spatial distribution functions of the center Pyr_14_^+^, anions (red), cations (blue), and Li^+^ (silver). The left image and the right image refer to the system including DCA^−^ and TCM^−^, respectively. f) Refer to the spatial distribution function of the central anion. The color scheme of this image is the same as panel e). c‐f) Reproduced with permission.^[^ ^26^ ^]^ Copyright 2021, Wiley‐VCH.

Characterization and electrochemical measurement tend to focus on the description of the phenomenon level of the results. Many strategies also require theoretical analysis to understand its in‐depth mechanism. The simulation and calculation can strengthen the insight into the deposition of lithium, the diffusion and the solvation chemistry of Li^+^.^[^ ^166^, ^167^ ^]^ Understanding the deposition behavior of lithium is essential for improving the performance. Based on the phase field model, the deposition rate of lithium in the conductive 3D host was revealed. In the early stage of deposition, the deposition rate of lithium was limited by electron transport. Hence, large *S*_A_ (*S*_current collector total surface_/*S*_electrode_, that is, small channel width as shown in the **Figure** **11**a) was beneficial to the process. While in the later stage, the radial growth of lithium in the pores turned to upward growth, and the deposition rate was mainly determined by ionic transmission. Thus, small *S*_v_ (*S*_current collector total surface_/*V*_pores in electrode_) could increase the deposition capacity of lithium.^[^ ^144^ ^]^ The results can be used as a reference for future design of 3D current collector's pore structure. The lithium/electrolyte interface is another area that has been valued, and it is particularly critical in the research of solid‐state batteries. Using large‐scale molecular dynamics (MD) simulation, Yang et al.^[^ ^25^ ^]^ proposed an atomic model of lithium deposition/dissolution at the lithium/solid electrolyte interface. It was revealed that sluggish diffusion of Li^+^ at the interface is the reason for the generation of interface nanopores. Nanopores led to the increase of contact resistance at the interface and thus the degradation of battery performance. Furthermore, due to more rapid ion diffusion, the coherent interface was less likely to cause nanopores than the incoherent interface (Figure 11b). This research puts forward a new direction for the modification of lithium metal batteries and guides the engineering of the Li‐solid electrolyte (SE) interface. The structure of the solvated sheath affects the quality of the formed SEI, and the properties of different electrolyte systems can be predicted through simulation. Via classic molecular dynamics simulations, Karimi et al.^[^ ^26^ ^]^ studied the properties of 1‐butyl‐1‐methylpyrrolidinium dicyanamide (Pyr_14_DCA) and 1‐butyl‐1‐methylpyrrolidinium tricyanomethanide (Pyr_14_TCM), and their binary solutions with the respective Li salts. As a result, the DCA^−^ showed lesser stability compared with TCM^−^, which was due to that DCA^−^ approaches both cations (i.e., Pyr_14_^+^ and Li^+^) more closely, that is, being more exposed to the consequent radical reactions (Figure 11c–f). Thus the SEI forming on the anode of the lithium metal battery using LiDCA: Pyr_14_DCA electrolyte was probably thicker. The subsequent experimental results are consistent with the simulation. The calculation and simulation have become reliable partners of experiments. Especially when the experiment is constrained by conditions and difficult to implement, calculation and simulation can give optimal choices.^[^ ^167^ ^]^

## 5 Outlook

Scientists have been struggling with the problems of lithium anode in the last decades. And now this process is pushed forward by the urgent demand for high‐energy‐density energy storage devices. It is both an opportunity and a challenge for lithium metal batteries. The progress of researches on lithium anode in recent years has been reviewed in this paper. In the past four decades, various models (including Sand's time model, anodic electronic field model, diffusion flux model, etc.) have been proposed to help understand the fundamental mechanism of anode issues and guide the experiment. Strategies to stabilize lithium anode are based on the above mechanisms, and various components (current collector, SEI, electrolyte, and separator) related to the lithium anode are modified. Although breakthrough has been made in solving anodic problems, there are still many obstacles in this field, which limits the commercialization of lithium metal batteries in a short period.

1) The fundamental failure mechanism of lithium anode is not clear enough. For example, whether the real reason of lithium failure is dendrite or corrosion remains controversial. Because of the high activity of lithium, it is difficult to study the surface of fresh lithium, which is unstable under the characterization conditions. Via vitrifying electrolyte, cryogenic electron microscopy is applied to observe lithium metal in native state.^[^ ^168^ ^]^ More in situ detection methods are urgently needed to complete the theory about lithium anode to guide experiments.

2) Optimizing the compositions of electrolyte enables the in situ SEI with excellent properties to form, promoting lithium metal batteries achieving high CE and long lifespan. The position and type of organic electrolyte components’ substituent have influence on the composition of SEI. More experiments, theoretical calculations and simulations should be conducted to establish the relationship between the molecular structure of organic electrolyte components and the properties of formed SEI, so as to develop more favorable electrolyte components. As for the ex situ SEI, poor contact between the ex situ SEI and lithium metal anode can be improved by using immersing or in situ growth method, but big efforts are still needed to improve the CE of the battery with ex situ SEI. In addition, the development of protective layers that can form a coherent interface with lithium metal is a new direction. Such materials include ZnO, SiO_2_, Al_2_O_3_, TiO_2_, LiGaO_2_, etc.^[^ ^25^ ^]^

3) The host can both reduce the local current density and limit the volume change of anode. And some preparation processes of hosts, such as coiling and solution casting, meet the requirement of large‐scale industrial production. The pore structure design of the host is one of the focus of future host's design, which affects both rate performance and capacity of the battery. However, the low CE arising from the severe corrosion generated by large specific surface area of hosts obstacles the development of this strategy. It is impossible to solve all problems of lithium anode in a single strategy. Instead, the combination of diverse methods ultimately enable host become a practicable strategy. Thus, it may be a good solution to combine the additives or HCE with hosts to reduce the corrosion, dendrites, and volume expansion of lithium anode simultaneously.

4) Inorganic/polymer hybrid solid electrolyte is a promising method in stabilizing lithium anode. Combining merits of both inorganic and polymer solid electrolyte, the hybrid solid electrolyte performs high conductivity of Li^+^, reasonable rigidity and good compatibility of the electrolyte/electrode's interface. However, despite great progress achieved in improving the conductivity of solid electrolyte, there is still a huge gap between solid electrolyte and liquid electrolyte in ion conductivity. Great efforts are needed to continuously enhance the conductivity of solid electrolyte. For example, the conduction pathway of Li^+^ in solid electrolyte needs to be studied to provide theoretical basis for establishing the optimal conduction pathway of Li^+^ in electrolyte. Moreover, developing thin‐film solid electrolytes may be another solution to further improve the conductivity of solid electrolytes. But the thickness of thin film may weaken the effect of solid electrolytes to inhibit dendrites. Therefore, a balance between high ionic conductivity and suppressing dendrites needs to be found. Eventually, the clear process of dendritic formation in solid lithium metal batteries should be revealed.

5) Some strategies have been revealed beneficial to lithium anode, such as local HCEs, SHES effect. But these researches have exposed many problems, and a big gap between the results of modification using these strategies and practical target is revealed. For instance, the limited ion transport in local HCEs inhibit the rate performance of lithium metal battery. Wide exploration should be carried out to improve the conductivity of Li^+^ in LHCEs. Due to the lack of effective SEI, the lithium anode cannot be protected well. Therefore, the CEs of lithium metal batteries modified by SHES effect are still far from the satisfactory level. This problem would be resolved if the additive could shield Li^+^ and contribute to the formation of effective SEI simultaneously. The cation in the additive is responsible for SHES effect, while the anion has a great influence on properties of SEI, which needs to be explored widely.

6) Experimental conditions close to practical application should be widely used, and the gap between research and practical applications urgently needs to be narrowed. Mild experimental conditions, such as thick lithium sheets, excess electrolyte amount, and low current density, keep lithium metal batteries away from practical application. From the perspective of industrialization, limited experimental conditions has been taken seriously in some studies.^[^ ^88^ ^]^ However, in many typical laboratory tests, the lithium anodes are still too thick (100–400 µm)^[^ ^48^, ^69^, ^169^ ^]^ and the electrolyte amount is excess (50 µL or more in coin batteries).^[^ ^48^, ^170^ ^]^ Besides, to our knowledge, the coulombic efficiency of the lithium metal battery is always tested at relatively low current density (≤1 mA cm^−2^). Under such a mild condition, the corrosion problem of lithium metal is not easily exposed, which leads to the so‐called high CE. From the results of studies that use low N/P ratios, lean electrolyte and pouch battery in the tests, considerable gap between the electrochemical performance obtained and the demand for practical application is revealed. With the guarantee of considerable energy density, the lifespans of these lithium metal batteries are only tens of cycles. Furthermore, there are some new issues with practical lithium metal batteries. For instance, LiH observed in practical batteries leads to the pulverization of anode;^[^ ^17^ ^]^ the corrosion of lithium anodes during calendar ageing is observed for the first time by cryo‐electron microscopy;^[^ ^171^ ^]^ the external pressure management of the pouch battery has an effect on the improvement of battery's performance.^[^ ^16^ ^]^ These problems urgently need to arouse researchers’ extensive attention.

In all, although the studies on anode modification of lithium metal batteries have made some progress, various strategies have knotty issues in comprehensively improving the performance of lithium metal batteries. And there is still a big gap between the electrochemical properties of lithium metal battery and practical application. Breakthroughs in this field are required to help revive lithium anodes.

## Conflict of Interest

The authors declare no conflict of interest.
